# Irisin Promotes Cardiac Homing of Intravenously Delivered MSCs and Protects against Ischemic Heart Injury

**DOI:** 10.1002/advs.202103697

**Published:** 2022-01-17

**Authors:** Wenjun Yan, Youhu Chen, Yongzhen Guo, Yunlong Xia, Congye Li, Yunhui Du, Chen Lin, Xiaoming Xu, Tingting Qi, Miaomiao Fan, Fuyang Zhang, Guangyu Hu, Erhe Gao, Rui Liu, Chunxu Hai, Ling Tao

**Affiliations:** ^1^ Department of Cardiology Xijing Hospital Fourth Military Medical University Xi'an 710032 China; ^2^ Beijing Anzhen Hospital Capital Medical University Beijing Institute of Heart, Lung and Blood Vessel Diseases Beijing 100029 China; ^3^ Center for Translational Medicine Temple University Philadelphia PA 19104 USA; ^4^ Department of Toxicology Shanxi Key Lab of Free Radical Biology and Medicine School of Public Health The Fourth Military Medical University Xi'an 710032 China

**Keywords:** CSF2RB, integrin *α*V/*β*5, irisin, ischemic heart injury, mesenchymal stromal cells

## Abstract

Few intravenously administered mesenchymal stromal cells (MSCs) engraft to the injured myocardium, thereby limiting their therapeutic efficacy for the treatment of ischemic heart injury. Here, it is found that irisin pretreatment increases the cardiac homing of adipose tissue‐derived MSCs (ADSCs) administered by single and multiple intravenous injections to mice with MI/R by more than fivefold, which subsequently increases their antiapoptotic, proangiogenic, and antifibrotic effects in rats and mice that underwent MI/R. RNA sequencing, Kyoto Encyclopedia of Genes and Genomes (KEGG) signaling pathway analysis, and loss‐of‐function studies identified CSF2RB as a cytokine receptor that facilitates the chemotaxis of irisin‐treated ADSCs in the presence of CSF2, a chemokine that is significantly upregulated in the ischemic heart. Cardiac‐specific CSF2 knockdown blocked the cardiac homing and cardioprotection abilities of intravenously injected irisin‐treated ADSCs in mice subjected to MI/R. Moreover, irisin pretreatment reduced the apoptosis of hydrogen peroxide‐induced ADSCs and increased the paracrine proangiogenic effect of ADSCs. ERK1/2‐SOD2, and ERK1/2‐ANGPTL4 are responsible for the antiapoptotic and paracrine angiogenic effects of irisin‐treated ADSCs, respectively. Integrin *α*V/*β*5 is identified as the irisin receptor in ADSCs. These results provide compelling evidence that irisin pretreatment can be an effective means to optimize intravenously delivered MSCs as therapy for ischemic heart injury.

## Graphical abstract

Irisin activates ERK1/2 in ADSCs by binding to integrin *α*V/*β*5, and increases CSF2RB, SOD2, and ANGPTL4 expression. The ischemic heart releases the chemokine CSF2 and forms a CSF2 gradient. CSF2RB responds to the CSF2 gradient and promotes the cardiac homing of ADSCs. SOD2 reduces ADSC apoptosis. ANGPTL4 is released from ADSCs and contributes to the paracrine proangiogenic effect of ADSCs.

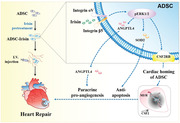

## 1 Introduction

Mesenchymal stromal cell (MSC) therapy has been developed as a treatment option for acute myocardial infarction (AMI) or heart failure (HF) caused by ischemic or nonischemic cardiomyopathy.^[^ ^1^ ^]^ However, a major factor limiting the benefits of cell therapy is the poor engraftment of the cells, which disappear rapidly after transplantation regardless of the cell type utilized. Several strategies, such as gene modification, hypoxic preconditioning, combined biomaterials, and simple repeated administration, have been developed to overcome poor engraftment. Accumulating studies support the concept that the repeated administration of cells can substantially increase their efficacy for ischemic heart repair.^[^ ^2^, ^3^ ^]^ Intramyocardial, intravenous, and intracoronary implantations are the most common methods for cell delivery. However, the safety and feasibility of repeated intramyocardial injection and intracoronary injection are limited.

The intravenous delivery of MSCs to patients recovering from MI is an attractive noninvasive strategy that allows for the repeated administration of large numbers of cells. Intravenous MSC delivery has been investigated in small and large animal models. With rare exceptions, modest beneficial effects of intravenous cell therapy on cardiac function have been observed in a variety of experimental settings (AMI, chronic MI, and nonischemic cardiomyopathy).^[^ ^4^ ^]^ However, all available studies indicate that after intravenous administration, the fraction of MSCs that home to the heart is low (≤3% of the total number injected within 24 h) and that this number becomes essentially zero by 7 days. Therefore, novel strategies that increase the cardiac homing of intravenously delivered MSCs and elucidation of the underlying mechanisms would obviate the need for the direct delivery of cells to the heart, making cell therapy simpler, cheaper, safer, and more broadly available.

Physical activity, widely used as a key component of strategies aimed at reducing post‐MI remodeling and attenuating HF progression in experimental animals, is recommended for patients who survive MI.^[^ ^5^, ^6^ ^]^ Exercise training has been shown to mobilize endogenous stem/progenitor cells for heart repair, but the mechanisms are still unclear.^[^ ^7^, ^8^, ^9^ ^]^ Physical activity stimulates the production of several hormone‐like molecules from skeletal muscle, termed “myokines”.^[^ ^10^, ^11^ ^]^ Irisin was identified as a myokine released into the circulation upon physical exercise that can stimulate adipocyte browning and thermogenesis in mice and humans.^[^ ^12^, ^13^ ^]^ Irisin is produced after the exercise‐induced promotion of the splicing or cleaving of the myocyte extracellular domain fibronectin type III domain‐containing protein 5 (FNDC5), a membrane integrated precursor protein controlled by myocyte peroxisome proliferator‐activated receptor‐*γ* coactivator 1*α*.^[^ ^14^ ^]^ However, whether irisin affects MSC‐based therapy for myocardial repair is largely unknown.

Thus, the aims of this study were to determine whether the myokine irisin regulates the cardiac homing and survival of intravenously infused adipose tissue‐derived MSCs (ADSCs) and their cardioprotective effects and to determine the mechanisms underlying this phenomenon, including the specific receptor(s) and intracellular signaling pathways involved.

## 2 Results

### 2.1 Exogenous Irisin Pretreatment Increases the Cardiac Engraftment of Intravenously Delivered ADSCs

The surface marker expression and differentiation potential of passage 2 ADSCs were assessed,^[^ ^15^, ^16^ ^]^ and these cells were used for subsequent experiments. To determine whether irisin affects the cardiac homing of intravenously injected ADSCs, we pretreated mouse ADSCs (mADSCs) with irisin or vehicle for 2 days. ADSCs were labeled with the lipophilic dye CM‐DiI immediately before intravenous injection.^[^ ^16^ ^]^

**Figure 1:**
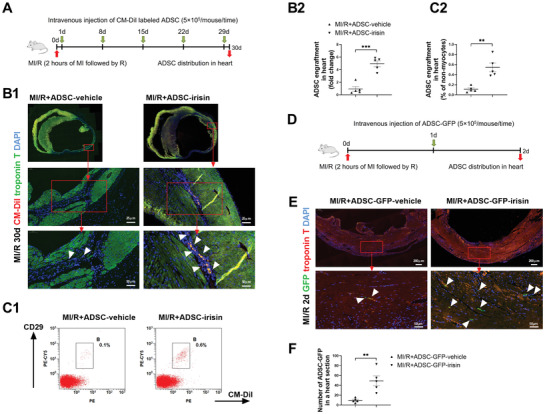
Irisin increases the cardiac engraftment of intravenously delivered ADSCs. A) Experimental scheme and timeline for cardiac homing studies using CM‐DiI‐labeled ADSC‐vehicle and ADSC‐irisin. At 1, 8, 15, 22, and 29 days after the MI/R operation, 5 × 10^5^ CM‐DiI+ ADSCs were infused intravenously via the vena angularis. B1) Representative images of CM‐DiI‐labeled ADSCs in hearts 30 days after MI/R. Heart tissue was immunostained for troponin T (green) and DAPI (blue). Engrafted ADSCs are CM‐DiI positive (white triangles). B2) Quantification of CM‐DiI‐labeled ADSCs in the heart sections (*n* = 5 mice per group). C1) Flow cytometric analysis of CM‐DiI‐labeled ADSCs in hearts. C2) Cardiac homing of ADSCs was quantified as the ratio of CM‐DiI+/CD29+ cells to the total number of nonmyocytes (*n* = 5 mice). D) Experimental scheme and timeline for cardiac homing studies using ADSCs isolated from EGFP transgenic mice (ADSC‐GFP). At 1 day after MI/R operation, 5 × 10^5^ ADSC‐GFP‐vehicle or ADSC‐GFP‐irisin were infused intravenously via the vena angularis. E) Representative images of ADSC‐GFP in hearts 2 days after MI/R. Heart tissue was immunostained for troponin T (red) and DAPI (blue). Engrafted ADSCs are GFP positive (white triangles). F) Quantification of ADSCs in the peri‐infarct area was determined by the number of ADSC‐GFP in a representative section (*n* = 5 mice per group). The data were analyzed by unpaired, 2‐tailed Student's *t* test. ADSC‐vehicle, ADSCs treated with vehicle; ADSC‐irisin, ADSCs treated with irisin (100 ng mL^−1^) for 2 days. **p < 0.01, ***p < 0.001.

C57BL/6J mice were subjected to myocardial ischemia‐reperfusion (MI/R) and then administered either vehicle‐treated ADSCs (ADSC‐vehicle, intravenous injection of 5 × 10^5^ ADSC‐vehicle at 1, 8, 15, 22, and 29 days after the MI/R operation) or irisin‐treated ADSCs (ADSC‐irisin). Immunostaining and flow cytometry assays were performed to detect CM‐DiI‐labeled ADSCs in the heart, lung, spleen, and liver at 30 days after MI/R (**Figure** **1**A; Figure S1A, Supporting Information). The immunofluorescence staining of tissue sections showed that vehicle‐treated ADSCs were mainly localized in the lung and spleen tissues and rarely localized in the heart and liver (Figure 1B; Figure S1B–E, Supporting Information). However, the cardiac engraftment of irisin‐treated ADSCs into mice post MI/R was significantly enhanced compared to that of vehicle‐treated ADSCs, while CM‐DiI‐labeled ADSCs predominantly engrafted in the ischemic (troponin T negative) region (Figure 1B). To better quantify the homing/retention of transplanted ADSCs, we digested the hearts after MI/R. Consistently, flow cytometric analyses of single‐cell nonmyocyte suspensions (Figure 1C) showed significantly more CM‐DiI^+^/CD29^+^ double‐positive cells in the ischemic myocardia of the MI/R+ADSC‐irisin group than in those of the MI/R+ADSC‐vehicle group. However, irisin pretreatment did not significantly alter the lung, spleen, or liver distribution of intravenously injected ADSCs (Figure S1B–E, Supporting Information).

To further examine whether irisin increases the cardiac homing of intravenously injected ADSCs, we isolated ADSCs from transgenic mice expressing enhanced green fluorescent protein (EGFP), treated them with vehicle or irisin for 2 days (ADSC‐GFP‐vehicle or ADSC‐GFP‐irisin), and then directly injected the cells 1 day after the MI/R operation (Figure 1D). Immunostaining was used to assess ADSC‐GFP in the heart at 2 days after MI/R. As shown in Figure 1E,F, irisin significantly increased the cardiac homing of ADSC‐GFP administered by a single injection. Next, we performed the in vivo competitive homing assay in mice 1 day post‐MI/R. Vehicle‐treated ADSCs (ADSC‐vehicle) were labeled with DiO (a green dye), and irisin‐treated ADSCs (ADSC‐irisin) were labeled with CM‐DiI (a red dye). An equal number of ADSC‐vehicle‐DiO and ADSC‐irisin‐DiI were then mixed before they were intravenously injected into the post‐MI/R mice. An aliquot of the cell mixture was kept and analyzed by flow cytometry (Figure S2A, Supporting Information). Flow cytometry assays were performed to detect ADSC‐vehicle‐DiO and ADSC‐irisin‐DiI in the “myocyte‐depleted” cardiac cell population and single‐cell suspensions of lung and spleen. Consistent with the data of the immunofluorescence staining of tissue sections, the intravenously injected ADSC‐vehicle‐DiO were mainly localized in the lung. The engraftment of ADSC‐irisin‐DiI in the lung and spleen was comparable to that of ADSC‐vehicle‐DiO (Figure S2B–E, Supporting Information). However, the cardiac homing of ADSC‐irisin‐DiI was increased fivefold over that of ADSC‐vehicle‐DiO (Figure S2F,G, Supporting Information). Taken together, these results demonstrated that exogenous irisin pretreatment specifically increased the cardiac engraftment of intravenously delivered ADSCs.

### 2.2 Exogenous Irisin Pretreatment Enhances the Cardioprotection of Intravenously Delivered ADSCs

**Figure 2:**
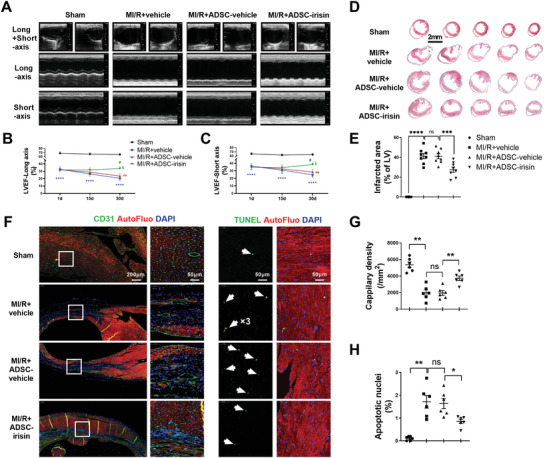
Irisin enhances the post‐MI/R cardioprotection of intravenously delivered ADSCs. A) Representative long‐axis and short‐axis M‐mode echocardiographic images of mice 30 days after MI/R. B) LVEF evaluated by long‐axis M‐mode echocardiography. C) LVEF evaluated by short‐axis M‐mode echocardiography. *n* = 10, 20, 22, and 24. The data were analyzed by 1‐way ANOVA, followed by a Bonferroni post hoc test. ****p < 0.0001 versus the Sham group, ^#^ *p* < 0.05 versus the MI/R+vehicle group, ^$^ *p* < 0.05 versus the MI/R+ADSC‐vehicle group. D) Five sections of representative Masson trichrome staining. E) Quantification of the fibrotic area 30 days after MI/R (*n* = 8 mice). The data were analyzed by Kruskal–Wallis tests, followed by Dunn post hoc tests. F) Representative images of capillary density determined by CD31 immunofluorescence staining (left, green) and TUNEL‐positive cardiomyocytes (right, green, indicated by the white arrows) in the ischemic area 30 days after MI/R. Cell nuclei were stained with DAPI (blue). G) Quantification of capillary density (*n* = 6 mice). The data were analyzed by 1‐way ANOVA, followed by a Bonferroni post hoc test. H) Quantification of TUNEL‐positive cardiomyocytes (*n* = 6 mice). The data were analyzed by Kruskal–Wallis test followed by Dunn post hoc test. ADSC‐vehicle, ADSCs treated with vehicle; ADSC‐irisin, ADSCs treated with irisin (100 ng mL^−1^) for 2 days. AutoFluo, autofluorescence. *p < 0.05, **p < 0.01, ***p < 0.001, ****p < 0.0001; ns, not significant.

Next, we assessed the cardioprotective effects of intravenously delivered ADSCs on mice post MI/R. ADSCs isolated from donor C57BL/6J mice were treated with irisin or vehicle for 2 days. Another group of C57BL/6J mice was randomly subjected to the following four treatments: Sham, MI/R+vehicle, MI/R+ADSC‐vehicle, or MI/R+ADSC‐irisin. Cardiac function was determined by consecutive echocardiography in both the long‐ and short‐axis M‐modes. The initial cardiac dysfunction was similar in all groups on day 1 after MI/R, as evidenced by the left ventricle ejection fraction (LVEF; **Figure** **2**A–C). Multiple intravenous deliveries of vehicle‐treated ADSCs did not significantly improve the cardiac function compared to that in the MI/R+vehicle group, as evidenced by the LVEF values at 15 and 30 days after MI/R (Figure 2A‐C). In contrast, ADSC‐irisin treatment significantly increased the LVEF (at 30 days) compared with that in the MI/R+ADSC‐vehicle group (Figure 2A–C).

To investigate the therapeutic mechanism(s) by which ADSC‐irisin promotes cardiac repair, we measured the degrees of fibrosis, cardiomyocyte apoptosis, and vascular density in the infarct border area of the heart 30 days post MI/R. The mice in the MI/R+ADSC‐irisin group showed significantly reduced infarct areas compared with those of MI/R+ADSC‐vehicle group mice (*p* < 0.05; Figure 2D,E). The vascular density in the infarct area border zone was significantly higher in the MI/R+ADSC‐irisin group than in the MI/R+ADSC‐vehicle group (Figure 2F,G). Moreover, the number of apoptotic cardiomyocytes was significantly decreased in the MI/R+ADSC‐irisin group compared with the MI/R+ADSC‐vehicle group (Figure 2F,H).

To exclude the potential heterogeneity of ADSCs between mice and rats, we next used a Sprague‐Dawley rat MI/R model to assess the cardiac homing and cardioprotective effects of rat ADSCs, which were isolated from transgenic rats expressing tdTomato (ADSC‐Tomato) and then treated with irisin (ADSC‐Tomato‐irisin) or vehicle (ADSC‐Tomato‐vehicle) for 2 days. The rat experimental protocol was the same as that for mice except that 5 × 10^6^ cells were delivered each time (Figure S3A, Supporting Information). Irisin pretreatment significantly increased the cardiac distribution of intravenously injected ADSC‐Tomato (Figure S3B,C, Supporting Information). Multiple ADSC‐Tomato‐vehicle administrations failed to significantly increase the LVEF (*p* > 0.05) compared with that achieved with vehicle. However, ADSC‐Tomato‐irisin significantly increased the LVEF at 30 days post MI/R compared with ADSC‐Tomato‐vehicle (Figure S3D–F, Supporting Information). Multiple intravenous injections of ADSC‐Tomato‐irisin also exerted an antifibrotic effect in rats that underwent MI/R (Figure S3G,H, Supporting Information).

Collectively, these in vivo studies provide clear evidence that multiple intravenous deliveries of irisin‐treated ADSCs promote post‐MI/R cardiac repair via antiapoptotic, proangiogenic, and antifibrotic mechanisms.

### 2.3 Irisin Increases CSF2RB Protein Expression in ADSCs in Response to the Chemokine CSF2

**Figure 3:**
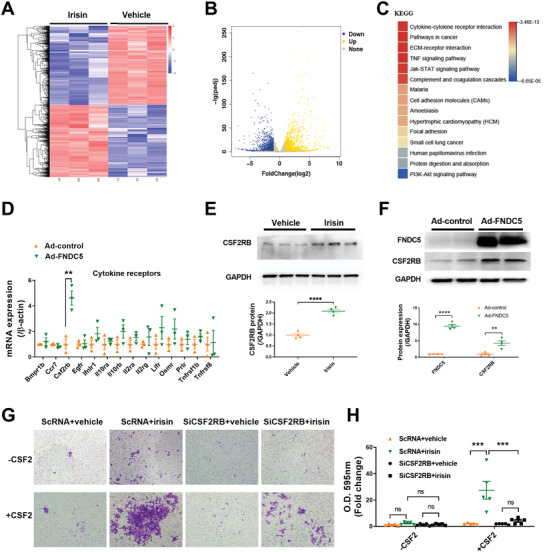
Irisin increases CSF2RB protein expression in ADSCs in response to the chemokine CSF2. A) Cluster analysis of differentially expressed genes determined by RNAseq of ADSCs treated with irisin (100 ng mL^−1^) and vehicle for 24 h. B) Volcano plot of RNAseq of ADSCs with irisin and vehicle treatments. The blue dots represent genes with downregulated expression induced by irisin with a value of log2FoldChange<‐1. The yellow dots represent genes with upregulated expression induced by irisin with a value of log2FoldChange>1. C) The top 15 KEGG pathways in ADSCs that were significantly affected by irisin. D) Real‐time PCR analysis of mRNA expression of cytokine receptors (14 in total) in the cytokine‐cytokine receptor pathway (*n* = 3). E) Western blots and quantification of CSF2RB protein expression in ADSC cell lysates (*n* = 4). ADSCs were treated with irisin (100 ng mL^−1^) or vehicle for 48 h. F) Western blots and quantification of FNDC5 and CSF2RB protein expression in ADSC lysates (*n* = 4). ADSCs were transfected with adenovirus‐FNDC5 (Ad‐FNDC5) or ADSC‐control (Ad‐control) for 48 h. G) Matrigel‐coated Transwell assay of ADSC chemotaxis to CSF2. H) The number of cells that had migrated to the lower chamber. ADSCs were transfected with scRNA or siRNA against CSF2RB (siCSF2RB) for 36 h and were treated with or without irisin for another 48 h. ADSCs were seeded in the upper chamber of the Transwell system. The Transwell system was placed in medium with or with CSF2 (100 ng mL^−1^) for 24 h. The data in (D), (E), and (F) were analyzed by unpaired, 2‐tailed Student's *t* test. The data in (H) were analyzed by 1‐way ANOVA, followed by Bonferroni post hoc test. **p < 0.01, ***p < 0.001, ****p < 0.0001; ns, not significant.

To explore the molecular mechanisms by which irisin pretreatment increases the cardiac homing of ADSCs, we used the nonbiased RNA sequencing (RNAseq) approach to analyze vehicle‐ and irisin‐treated rADSCs. Up to 1155 genes in rADSCs showed significant upregulation after irisin treatment (**Figure** **3**A,B). Kyoto Encyclopedia of Genes and Genomes (KEGG) signaling pathway analysis identified cytokine‐cytokine receptor interaction as the most significantly enriched signaling pathway (Figure 3C). Irisin significantly increased the expression of 26 cytokines and 19 cytokine receptors in the cytokine‐cytokine receptor interaction pathway (Table I, Supporting Information). These results suggest that irisin promotes the cardiac homing of ADSCs by classic cytokine‐cytokine receptor‐mediated chemotaxis.

Since MSCs express cytokine receptors in response to the cytokine gradient in the ischemic heart,^[^ ^17^ ^]^ we focused on cytokine receptors. To identify the specific cytokine receptor(s) that contribute to the cardiac homing of irisin‐treated ADSCs, we increased the endogenous irisin concentration in rADSCs via transfection with an adenovirus harboring FNDC5 (Ad‐FNDC5) (Figure S4, Supporting Information). Among the abovementioned 19 cytokine receptors that were significantly increased by exogenous irisin in rADSCs (Table S1, Supporting Information), 14 genes in rADSCs were detected by quantitative PCR (Figure 3D). FNDC5 overexpression significantly increased the mRNA expression of Csf2rb but not that of the other 13 genes (Figure 3D). More importantly, both irisin treatment and FNDC5 overexpression significantly increased the protein expression of CSF2RB in rADSCs (Figure 3E,F).

CSF2RB is a common subunit of CSF2R, the receptor for CSF2 (also named GM‐CSF). CSF2 is a chemokine that plays an important role in the mobilization and homing of inflammatory cells to infarcted heart tissue.^[^ ^18^, ^19^ ^]^ We performed an in vitro Transwell migration assay to assess the effects of irisin on the chemotaxis of ADSCs toward a CSF2 gradient. The migration of ADSCs pretreated with irisin for 2 days toward CSF2 was significantly enhanced (Figure 3G,H). However, irisin did not significantly alter ADSC migration in the absence of a CSF2 gradient. More importantly, CSF2RB knockdown in ADSCs largely blocked the chemotactic movement of ADSC‐irisin toward CSF2 (Figure S5A,B, Supporting Information; Figure 3G,H).

Next, we performed an in vivo study to investigate whether CSF2RB is essential for the irisin‐increased cardiac homing of intravenously delivered ADSCs. ADSCs were treated with irisin after they were transfected with scRNA (ADSC‐irisin‐CSF2RB^WT^) or siRNA against CSF2RB (ADSC‐irisin‐CSF2RB^KD^). Consistent with the data in Figure 1E,F, irisin significantly increased the cardiac homing of ADSCs administered by a single injection. However, the loss of CSF2RB in ADSCs before irisin administration significantly reduced the number of CM‐DiI+ ADSCs in heart tissue (Figure S5C,D, Supporting Information). Collectively, these results demonstrate that irisin increases the CSF2RB protein expression in ADSCs, which facilitates their response to the chemokine CSF2 and may increase their cardiac homing after intravenous injection.

### 2.4 Myocardial CSF2 is Essential for the Cardiac Homing and Cardioprotective Abilities of Intravenously Delivered ADSCs Pretreated with irisin

**Figure 4:**
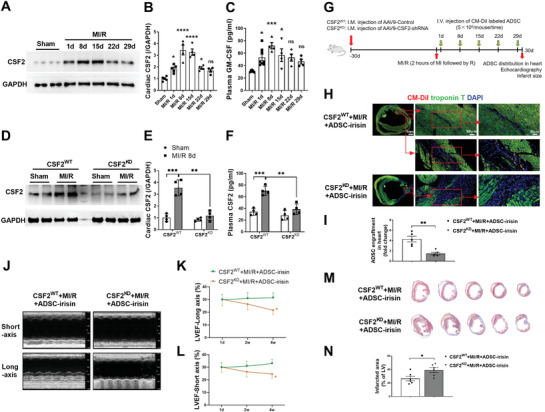
Myocardial CSF2 is essential for cardiac homing and cardioprotection of intravenously delivered irisin‐pretreated ADSCs. A,B). Western blots (A) and quantification (B) of CSF2 protein expression in the mouse infarct border zone 1, 8, 15, 22, and 29 days after MI/R. *n* = 4–5 mice. C) Plasma CSF2 was determined by ELISAs of mice in the Sham and MI/R groups. *n* = 4–8 mice. *p < 0.05, ***p < 0.001, ****p < 0.0001 vs the Sham group. ns, not significant. D,E) Western blots (D) and quantification (E) of CSF2 protein expression in the mouse infarct border zone 8 days after MI/R. Mice were intramyocardially injected with AAV9‐control and AAV9‐CSF2‐shRNA 1 month before MI/R. *n* = 4 mice. F) Plasma CSF2 was determined by ELISAs 8 days after MI/R. *n* = 4 mice. **p < 0.01, ***p < 0.001. ns, not significant. G) Experimental scheme and timeline for cardiac CSF2 loss‐of‐function studies. AAV9‐CSF2‐shRNA or AAV9‐control were intramyocardially (I.M.) injected into three sites in the left ventricle. At 1, 8, 15, 22, and 29 days after the MI/R operation, 5 × 10^5^ CM‐DiI+ ADSC‐irisin were infused intravenously via the vena angularis. ADSC‐irisin, ADSCs treated with irisin (100 ng mL^−1^) for 48 h. H) Representative images of CM‐DiI‐labeled ADSCs in hearts 30 days after MI/R. Heart tissue was immunostained for troponin T (green) and DAPI (blue). Engrafted ADSCs are CM‐DiI positive (red). I) Quantification of CM‐DiI‐labeled ADSCs in the peri‐infarct area was determined by the percentage of red area in a representative section (*n* = 5 mice per group). **p < 0.01. J) Representative long‐axis and short‐axis M‐mode echocardiographic images 30 days after MI/R. K) LVEF evaluated by long‐axis M‐mode echocardiography. L) LVEF evaluated by short‐axis M‐mode echocardiography. *n* = 12, 10. *p < 0.05. M) Five sections of representative Masson trichrome staining. N) Quantification of the fibrotic area 30 days after MI/R (*n* = 6 mice). *P < 0.05. The data in (B) and (C) were analyzed by 1‐way ANOVA, followed by a Bonferroni post hoc test. Others were analyzed by unpaired, 2‐tailed Student's *t* test.

Our in vitro studies showed that irisin enhanced the ability of ADSCs to respond to CSF2‐induced chemotaxis by upregulating CSF2RB expression, and the roles of CSF2/CSF2RB in the homing and cardioprotection of MSCs have never been reported. We found that CSF2 was upregulated in both the heart tissues and plasma of mice subjected to MI/R at multiple time points, peaking at 8 days (**Figure** **4**A–C). To determine whether the upregulated protein expression of CSF2RB contributed to the enhanced cardiac homing ability of ADSC‐irisin, we performed a CSF2 loss‐of‐function study. We designed three short hairpin RNAs (shRNAs) targeting CSF2 and found that one of them efficiently inhibited CSF2 mRNA and protein expression in cardiac fibroblasts (Figure S6A–C, Supporting Information), the heart cell type expressing the highest levels of CSF2.^[^ ^18^ ^]^ We then packed the CSF2 shRNA into adeno‐associated virus serotype‐9 (AAV9‐CSF2‐shRNA) and intramyocardially injected mice with AAV9‐CSF2‐shRNA (CSF2^KD^ mice) or AAV9‐control (CSF2^WT^ mice). After 30 days, both the CSF2^WT^ and CSF2^KD^ mice were subjected to MI/R. Cardiac tissue and plasma CSF2 expression was significantly inhibited in the CSF2^KD^+MI/R group at 8 days post MI/R compared to that in the CSF2^WT^+MI/R group (Figure 4D–F). We then delivered irisin‐treated ADSCs to mice in the CSF2^WT^+MI/R and CSF2^KD^+MI/R groups intravenously multiple times (Figure 4G). The ability of irisin‐treated ADSCs to home to the hearts of CSF2^KD^+MI/R mice was significantly reduced compared to that of the cells homing to the CSF2^WT^+MI/R mouse hearts at 30 days after MI/R (Figure 4H,I). Consistent with the reduced ADSC homing, the CSF2^KD^+MI/R mice showed significantly lower LVEF values (Figure 4J–L) and significantly higher infarcted areas (Figure 4M,N) than the CSF2^WT^+MI/R controls after ADSC‐irisin injection. Next, we intravenously injected ADSC‐irisin with IgG or a blocking antibody (BA) against CSF2 in an in vivo study and tested CM‐DiI‐labeled ADSCs in the heart 1 day after intravenous injection (2 days after MI/R). Compared with IgG, CSF2‐BA significantly inhibited the cardiac homing of ADSC‐irisin (Figure S5C,D, Supporting Information). Collectively, these studies demonstrate that irisin facilitates the cardiac homing of intravenously transplanted ADSCs via a CSF2/CSF2RB axis.

### 2.5 Irisin Pretreatment Protects ADSCs against Apoptosis and Enhances Their Ability to Increase Angiogenesis

**Figure 5:**
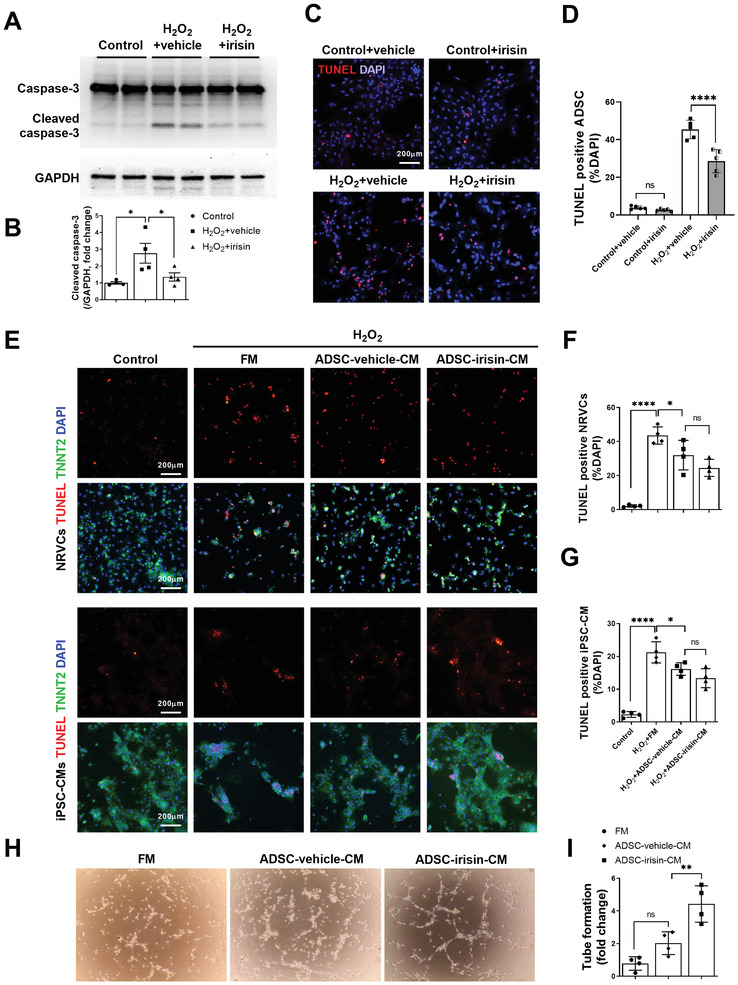
Irisin pretreatment protects ADSCs against apoptosis and enhances their ability to increase angiogenesis. A,B) Western blots and quantification of cleaved caspase‐3 protein expression in ADSCs. ADSCs were pretreated with or without irisin (100 ng mL^−1^, 48 h), washed to remove irisin, and then subjected to H_2_O_2_ (200 µm, 6 h). C) Representative images of TUNEL‐positive ADSCs (red) with or without H_2_O_2_ (200 µm, 6 h). Cell nuclei were stained with DAPI (blue). D) Quantification of TUNEL positive ADSCs (*n* = 5). E–G) Representative images of TUNEL‐positive (red) NRVCs and iPSC‐CMs. NRVCs and iPSC‐CMs were treated with FM, ADSC‐vehicle‐CM, or ADSC‐irisin‐CM 15 min before H_2_O_2_ (200 µm, 6 h) administration. NRVCs and iPSC‐CMs are TNNT2 positive (green). *n* = 4. H,I) The paracrine angiogenesis of ADSC‐vehicle‐CM and ADSC‐irisin‐CM was evaluated by rCAEC tube formation assays. *n* = 4. The data were analyzed by 1‐way ANOVA, followed by a Bonferroni post hoc test. *p < 0.05, **p < 0.01, ****p < 0.0001; ns, not significant.

To better understand the cardioprotective effects of irisin‐treated ADSCs after they enter ischemic heart tissue, we performed in vitro studies. Cell Counting Kit‐8 assays demonstrated that irisin did not significantly alter the ADSC growth rate (Figure S6D, Supporting Information). However, irisin pretreatment significantly decreased ADSC apoptosis upon exposure to hydrogen peroxide (H_2_O_2_), as shown by the quantification of cleaved caspase‐3 (**Figure** **5**A,B) and TdT‐mediated dUTP nick‐end labeling (TUNEL) (Figure 5C,D). Quantitative PCR demonstrated that irisin treatment for 7 days failed to affect the cardiogenic, vasculogenic, or fibrogenic differentiation of ADSCs (Figure S6E, Supporting Information).

Having demonstrated that the administration of irisin‐treated ADSCs significantly reduced cardiomyocyte apoptosis and increased angiogenesis in vivo, we next determined whether irisin could improve the paracrine functions of ADSCs. The effects of fresh medium (FM), conditioned medium (CM) from vehicle‐treated ADSCs (ADSC‐vehicle‐CM), and CM from irisin‐treated ADSCs (ADSC‐irisin‐CM) on H_2_O_2_‐induced cardiomyocyte apoptosis was determined. We used both primary neonatal rat ventricular cardiomyocytes (NRVCs) and human‐induced pluripotent stem cell‐derived cardiomyocytes (iPSC‐CMs). Compared with FM, ADSC‐vehicle‐CM significantly decreased the apoptosis of NRVCs and iPSC‐CMs (Figure 5E–G). However, ADSC‐irisin‐CM did not further reduce the apoptosis of NRVCs or iPSC‐CMs compared with that with ADSC‐vehicle‐CM. We then determined the effects of irisin on the paracrine proangiogenic effect of ADSCs. After the resuspension of rat coronary artery endothelial cells (rCAECs) in FM, ADSC‐vehicle‐CM, or ADSC‐irisin‐CM, tube formation assays were performed. Compared with ADSC‐vehicle‐CM, ADSC‐irisin‐CM significantly increased the tube formation ability of the cells (Figure 5H,I). Taken together, these in vitro studies demonstrated that irisin pretreatment improves the survival of ADSCs and enhances their paracrine proangiogenic effects but not their paracrine antiapoptotic effects.

### 2.6 Irisin Increases the ANGPTL4 and SOD2 Expression in ADSCs via Activating ERK1/2

**Figure 6:**
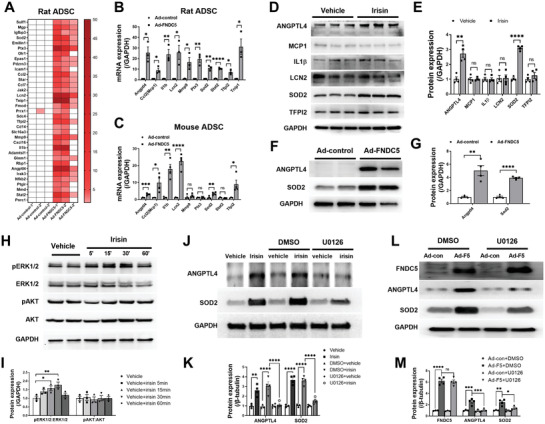
Irisin increases ANGPTL4 and SOD2 expression in ADSCs via ERK1/2 activation. A) Quantitative PCR analysis of mRNA expression for genes of interest (35 in total) in rat ADSCs transfected with adenovirus‐control (Ad‐control) or adenovirus‐FNDC5 (Ad‐FNDC5). These 35 genes were increased >2‐fold by FNDC5 overexpression. *n* = 3. B) Statistical significance of mRNA expression for the top ten genes of interest among the 35 genes in (A). C) Quantitative PCR analysis of mRNA expression for the ten genes in (B) in mouse ADSCs transfected with Ad‐control or Ad‐FNDC5 for 48 h. *n* = 4. D,E) Western blots and quantification of protein expression for genes of interest (6 in total) in ADSC lysates. ADSCs were treated with 100 ng mL^−1^ irisin or vehicle for 48 h. *n* = 4. F,G) Western blots and quantification of ANGPTL4 and SOD2 protein expression in rat ADSCs transfected with Ad‐control or Ad‐FNDC5 for 48 h. *n* = 4. H,I) Western blots and quantification of phosphorylated ERK1/2 and AKT in ADSCs after 100 ng mL^−1^ irisin treatment for 5, 15, 30, and 60 min (*n* = 3). *p < 0.05. J,K) Western blots and quantification of ANGPTL4 and SOD2 in ADSC cell lysates demonstrated that U0126 (an ERK1/2 activation inhibitor) blocked irisin‐induced increases in ANGPTL4 and SOD2 protein expression (U0126: 10 µm, 2 h before irisin treatment). *n* = 4. L,M) Western blots and quantification of FNDC5, ANGPTL4, and SOD2 in rat ADSCs transfected with Ad‐con or Ad‐F5 with or without U0216 for 48 h. *n* = 4. The data in (I), (K), and (M) were analyzed by 1‐way ANOVA, followed by Bonferroni post hoc test. Others were analyzed by unpaired, 2‐tailed Student's *t* test.

To identify the molecular mechanisms by which irisin affects ADSC survival and paracrine function, we reanalyzed the RNAseq data (Figure 3A,B). The ADSC‐irisin profile revealed that 1155 genes were significantly upregulated (Figure 3A,B), among which 141 genes in the ADSC‐vehicle group had a mean count of >1000 (Table S2, Supporting Information). We then narrowed down these genes in rADSCs overexpressing FNDC5. Among these 141 genes, 132 were detectable in rADSCs by quantitative PCR (Figure S7A, Supporting Information), and 35 were increased by >2‐fold upon FNDC5 overexpression (**Figure** **6**A). We focused on the top 10 of these 35 genes (Angptl4, Ccl2 (Mcp1), Il1b (IL1*β*), Lcn2, Mmp9, Ptx3, Sod2, Stat2, Tfpi2, and Tnipl) (Figure 6B). However, Tnipl was not detectable in mADSCs, while Mmp9, Ptx3, and Stat2 were not significantly altered by FNDC5 overexpression (Figure 6C). Western blot analysis confirmed that irisin significantly increased the protein expression of SOD2 and ANGPTL4 but not that of MCP1, IL1*β*, LCN2, or TFPI2 in rADSCs (Figure 6D,E). FNDC5 overexpression also significantly increased the SOD2 and ANGPTL4 expression in rADSCs (Figure 6F,G).

We next aimed to identify the signaling molecules upstream of the ANGPTL4 and SOD2 proteins in the presence of FNDC5/irisin. Because the activation of AKT and ERK1/2 was shown to be involved in the effects of irisin on downstream signaling,^[^ ^11^, ^20^ ^]^ we quantified both AKT and ERK1/2 phosphorylation after irisin treatment for different amounts of time. ERK1/2 phosphorylation was initially increased after 5 min and was significantly increased after 15–30 min of incubation with irisin (Figure 6H–I). AKT phosphorylation was not altered during 60 min of irisin stimulation. To identify a cause‐effect relationship between ERK1/2 activation and ANGPTL4/SOD2 protein expression after irisin treatment, we administered U0126 (an ERK1/2 activation inhibitor, 10 µm) 2 h before irisin treatment. Irisin failed to increase ERK1/2 phosphorylation in the presence of U0126 (Figure S7B,C, Supporting Information). Importantly, the irisin‐induced upregulation of ANGPTL4 and SOD2 protein expression in rADSCs was almost completely blocked by U0126 (Figure 6J,K). U0126 also abolished the upregulation of ANGPTL4 and SOD2 protein expression in ADSCs overexpressing FNDC5 (Figure 6L,M). These results demonstrate that irisin upregulates ANGPTL4/SOD2 expression via ERK1/2 activation.

### 2.7 ERK1/2‐SOD2 and ERK1/2‐ANGPTL4 are Responsible for the Irisin‐Induced ADSC Antiapoptotic and Paracrine Angiogenic Effects, Respectively

**Figure 7:**
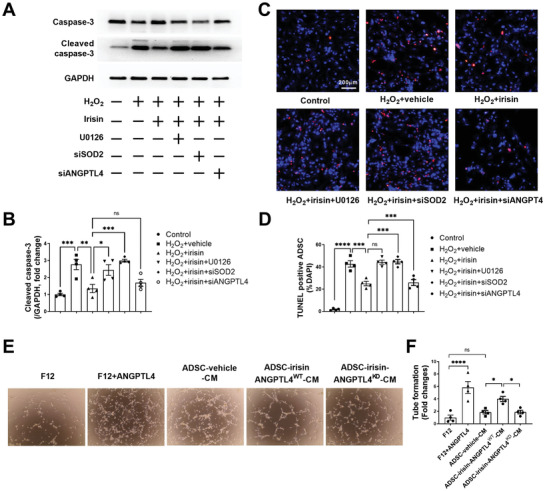
ERK1/2‐SOD2 and ERK1/2‐ANGPTL4 are responsible for the antiapoptotic and paracrine angiogenic effects of irisin‐induced ADSCs, respectively. A,B) Western blots and quantification of cleaved caspase‐3 protein expression in ADSCs. C) Representative images of TUNEL‐positive ADSCs (red) with or without H_2_O_2_ (200 µm, 6 h). Cell nuclei were stained with DAPI (blue). D) Quantification of TUNEL‐positive ADSCs. ADSCs were transfected with siRNA against SOD2 (siSOD2) or siRNA against ANGPTL4 (siANGPTL4) 36 h before irisin treatment (100 ng mL^−1^, 24 h). U0126: 10 µm, 2 h before irisin treatment. *n* = 4. E,F) Tube formation of rCAECs treated with F12, F12+ANGPTL4 (2 µg mL^−1^), ADSC‐vehicle‐CM, ADSC‐irisin‐ANGPTL4^WT^‐CM, or ADSC‐irisin‐ANGPTL4^KD^‐CM. *n* = 4. ANGPTL4^WT^, ANGPTL4 scRNA; ANGPTL4^KD^, ANGPTL4 siRNA. The data were analyzed by 1‐way ANOVA, followed by a Bonferroni post hoc test. *p < 0.05, **p < 0.01, ***p < 0.001, ***p < 0.0001. ns, not significant.

SOD2 is a mitochondrial antioxidant gene that has been shown to protect human MSCs from oxidative stress‐induced mitochondrial dysfunction.^[^ ^21^ ^]^ ANGPTL4, a secreted protein, has been implicated in mediating the paracrine function of MSCs.^[^ ^22^ ^]^ We next aimed to investigate the possible roles of ERK1/2‐SOD2 and ERK1/2‐ANGPTL4 in irisin‐mediated ADSC behaviors. The antiapoptotic effect of irisin on ADSCs was almost completely abolished by U0126, as evidenced by the quantification of cleaved caspase‐3 (**Figure** **7**A,B) and TUNEL staining (Figure 7C,D). Genetic knockdown of SOD2 by siRNA also significantly abolished the antiapoptotic effects of irisin on ADSCs (Figure S8A, Supporting Information; Figure 7A–D). However, siRNA‐mediated ANGPTL4 knockdown did not significantly alter the antiapoptotic effect of irisin on ADSCs (Figure S8B, Supporting Information; Figure 7A–D).

Because irisin increased ANGPTL4 expression, we treated NRVCs and rCAECs with recombinant ANGPTL4. ANGPTL4 did not significantly reduce H_2_O_2_‐induced NRVC apoptosis (Figure S8C,D, Supporting Information). However, ANGPTL4 significantly increased the tube formation ability of rCAECs compared with that induced by F12 (Figure 7E,F). Moreover, ANGPTL4 knockdown (ANGPTL4^KD^), but not SOD2 knockdown (SOD2 ^KD^), substantially inhibited the paracrine angiogenic effect of irisin‐treated ADSCs (Figure 7E,F; Figure S8E,F, Supporting Information). Collectively, these data demonstrate that the antiapoptotic effect of irisin is mediated by SOD2 upregulation and that the paracrine proangiogenic function of irisin‐treated ADSCs is mediated by ANGPTL4 release, followed by ERK1/2 activation.

### 2.8 Irisin Increases the ANGPTL4, CSF2RB, and SOD2 Expression in ADSCs via Integrin *α*V/*β*5

A previous study reported that lipid raft–mediated endocytosis plays an important role in facilitating the entry of irisin into mouse lung epithelial cells under I/R stress conditions.^[^ ^23^ ^]^ Our results showed that the increased mRNA expression of Angptl4, Csf2rb, and Sod2 induced by irisin was further increased instead of being blocked by nystatin, a known inhibitor of lipid raft–mediated endocytosis (Figure S9A–C, Supporting Information), suggesting that irisin activates its downstream signals by binding to its membrane receptor. Kim et al. first identified a subset of integrins, especially those associated with *α*V integrin, that function as irisin receptors in mouse osteocytes and fat tissues.^[^ ^11^ ^]^ These researchers found that integrin *α*V/*β*5 has the highest affinity for irisin and is required for the cellular response to irisin. Oguri et al. recently reported that the *α*V/*β*1 and *α*V/*β*5 integrins mediate the activation of integrin‐FAK signaling in response to irisin in a subset of mouse adipocyte progenitor cells.^[^ ^24^ ^]^

**Figure 8:**
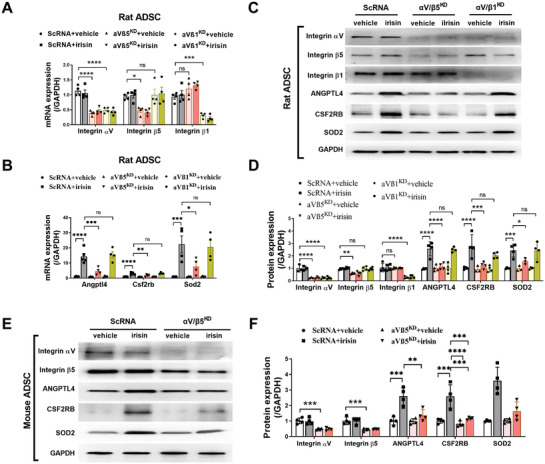
Irisin increases ANGPTL4, CSF2RB, and SOD2 expression in ADSCs via integrin *α*V/*β*5. A,B) Quantitative PCR analysis of mRNA expression of integrin *α*V, *β*5, and *β*1 (A) and Angptl4, Csf2rb, and Sod2 (B) in rat ADSCs. ADSCs were transfected with scRNA, siRNAs against integrin *α*V and *β*5 (*α*V*β*5^KD^), or siRNAs against integrin *α*V and *β*1 (*α*V*β*1^KD^) for 36 h and then treated with irisin (100 ng mL^−1^) for another 24 h. *n* = 3–4. C,D) Western blots and quantification of integrin *α*V, *β*5, and *β*1, ANGPTL4, CSF2RB, and SOD2 protein expression in rat ADSCs. ADSCs were transfected with scRNA, siRNAs against integrin *α*V and *β*5 (*α*V*β*5^KD^), or siRNAs against integrin *α*V and *β*1 (*α*V*β*1^KD^) for 36 h and then treated with irisin (100 ng mL^−1^) for another 48 h. *n* = 3–4. E,F) Western blots and quantification of integrin *α*V and *β*5, ANGPTL4, CSF2RB, and SOD2 protein expression in mouse ADSCs. ADSCs were transfected with scRNA or siRNAs against integrin *α*V and *β*5 (*α*V*β*5^KD^) for 36 h and then treated with irisin (100 ng mL^−1^) for another 48 h. *n* = 3–4. The data were analyzed by 1‐way ANOVA, followed by a Bonferroni post hoc test. *p < 0.05, **p < 0.01, ***p < 0.001, ****p < 0.0001; ns, not significant.

We analyzed the expression profiles of all integrins in both rADSCs and mADSCs, revealing the expression of 17 *α* subunits and 8 *β* subunits of the integrin family (Table S3, Supporting Information). Among the integrin *α* subunits, *α*V was expressed at relatively high levels in both rADSCs (ranked fifth) and mADSCs (ranked second). Integrin *β*1 showed the highest expression among all *β* subunits in both rADSCs and mADSCs. Integrin *β*5 ranked third in rADSCs and second in mADSCs. Based upon these data and previous reports,^[^ ^11^, ^24^ ^]^ we analyzed the involvement of the *α*V/*β*1 and *α*V/*β*5 integrins in the roles of irisin in rADSCs. We simultaneously knocked down integrin *α*V and integrin *β*5 in rADSCs using appropriate siRNAs (*α*V/*β*5^KD^) (**Figure** **8**A). The upregulated mRNA and protein expression of ANGPTL4, CSF2RB, and SOD2 by irisin was largely blocked in ADSCs with *α*V/*β*5^KD^ (Figure 8B–D). Irisin did not increase the ERK1/2 phosphorylation in ADSCs with *α*V/*β*5^KD^ (Figure S9D,E, Supporting Information). However, simultaneous knockdown of integrin *α*V/*β*1 (*α*V/*β*1^KD^) did not significantly alter the increased mRNA and protein expression of ANGPTL4, CSF2RB, and SOD2 induced by irisin (Figure 8A–D). Finally, we performed an integrin *α*V/*β*5 loss‐of‐function study in mADSCs. When integrin *α*V/*β*5 was knocked down in mADSCs, irisin did not increase the protein expression of ANGPTL4, CSF2RB, or SOD2 (Figure 8E,F). Taken together, these data identify integrin *α*V/*β*5, but not *α*V/*β*1, as a specific irisin receptor in ADSCs.

## 3 Discussion

Increasing preclinical evidence suggests that the intravenous administration of MSCs is effective for the structural and functional recovery of the infarcted heart.^[^ ^25^, ^26^, ^27^ ^]^ Accordingly, clinical trials are underway to determine the benefits of intravenous MSC‐based therapies.^[^ ^28^ ^]^ MSCs can migrate through the bloodstream to different organs under the guidance of chemical signaling‐related navigation, which facilitates their migration and engraftment to the ischemic myocardium and mediates repair. Although MSCs trapped in other organs (such as the lung and spleen) enhance cardiac repair by releasing anti‐inflammatory proteins,^[^ ^29^, ^30^ ^]^ active cell homing to the site of injury represents a critical step to the successful clinical use of intravenously delivered MSCs for myocardial repair.^[^ ^31^ ^]^ Because intravenous injection results in only a minuscule percentage of cells engrafting in the heart, the optimization of MSC homing efficiency is a major hurdle for this treatment.

In the present study, we made several novel observations. First, we demonstrated that irisin promotes the cardiac homing and cardioprotection of ADSCs administered via multiple intravenous injections. Clinical studies have shown that circulating irisin levels are lower in patients with either stable coronary artery disease or MI than in healthy controls^[^ ^32^ ^]^ and that lower irisin levels are an independent predictor of coronary artery disease severity.^[^ ^33^ ^]^ We demonstrated that irisin pretreatment increased the cardiac homing of intravenously injected ADSCs in post MI/R mice and rats by tracking ADSCs with CM‐DiI staining or genetic GFP and tdTomato labeling. Irisin specifically increased ADSC accumulation in the ischemic heart but not in the lung, spleen, or liver. Although the CM of ADSC‐vehicle significantly reduced cardiomyocyte apoptosis in the in vitro study, multiple intravenous injections of vehicle‐treated ADSCs failed to alter the cardiomyocyte apoptotic rate, infarct size, or LVEF in the in vivo study. The possible reason is that the majority of the intravenously injected ADSCs were distributed to other tissues; the paracrine factors secreted by ADSCs were heavily diluted in the blood stream and thus failed to provide protection for the ischemic heart tissue. However, multiple intravenous injections of irisin‐treated ADSCs significantly improved cardiac structure and function, as evidenced in both post‐MI/R mice and rats. Because intravenous transplantation is easy and safe for repeated injections, these results indicate that the intravenous administration of MSCs after irisin incubation may be more practical for patients with ischemic heart disease. Many studies have reported that irisin alone protects against ischemic heart injury by inhibiting cardiomyocyte apoptosis and promoting angiogenesis;^[^ ^34^, ^35^, ^36^ ^]^ the combination of MSC and irisin together is likely to exert a synergistic cardioprotection against ischemic heart disease.

Second, we identified CSF2/CSF2RB as a new chemotactic signaling axis in the cardiac homing of MSCs. The multifunctional growth factor CSF2 controls leukocyte production, proliferation, differentiation, and survival.^[^ ^18^ ^]^ Various cell types, such as macrophages, fibroblasts, endothelial cells, and B cells, produce CSF2.^[^ ^19^ ^]^ As an endogenous cytokine, CSF2 is actively secreted in response to various types of injuries, including MI.^[^ ^18^, ^19^, ^37^ ^]^ After MI, cardiac fibroblasts are the most abundant and nearly exclusive CSF2 producers.^[^ ^18^ ^]^ CSF2 enhances the migratory capacity of MSCs and their therapeutic effects in an endometrial ablation animal model.^[^ ^37^ ^]^ In the present study, we found that irisin directs the cardiac homing of intravenously delivered ADSCs by upregulating CSF2RB expression. When CSF2RB in ADSCs was knocked down, the chemotaxis of irisin‐treated ADSCs toward CSF2 was largely abolished. Moreover, CSF2 expression was increased in both the heart tissues and plasma of mice that underwent MI/R at multiple time points, peaking at 8 days. When CSF2 was knocked down in the ischemic heart or was inhibited by its blocking antibody, irisin‐treated ADSCs lost most of their homing capacity. The infarcted heart tissue with increased CSF2 expression recruits intravenously delivered irisin‐pretreated MSCs, which has a higher expression of CSF2RB. Other tissues are not able to recruit the MSCs from the blood because their CSF2 expression remains low after MI.^[^ ^18^ ^]^ These results support CSF2/CSF2RB as a potential therapeutic target to improve the cardiac homing of intravenously administered MSCs for the treatment of ischemic heart disease.

Third, we uncovered new cellular and molecular mechanisms that directly underlie the effects of irisin on ADSCs. After homing to the ischemic heart, the cells are in a harsh ischemic, hypoxic, and inflammatory microenvironment. The survival potency of transplanted MSCs largely determines their cardioprotective effects.^[^ ^38^ ^]^ FNDC5 and irisin exert antiapoptotic effects on many cell types, including cardiomyocytes and alveolar cells.^[^ ^23^, ^39^ ^]^ Based on RNAseq results, we focused on the top 10 genes among 35 genes that were increased by FNDC5/irisin and identified SOD2 as a potential antiapoptotic mediator. The superoxide dismutase (SOD) family is an important component of the antioxidant defense system, among which SOD2 is localized in mitochondria. We provided direct evidence that the antiapoptotic effect of irisin on ADSCs was mediated by the ERK1/2‐SOD2 pathway because ERK1/2 inactivation and SOD2 knockdown abolished the antiapoptotic effect.

Our in vivo studies showed that irisin‐treated ASDCs improved post‐MI/R cardiac remodeling and function. At the cellular level, irisin‐treated ASDCs increased the capillary density and inhibited cardiomyocyte apoptosis in the peri‐infarct area. Among many characteristics, direct transdifferentiation and paracrine effector release are the 2 most important mechanisms by which implanted stem cells protect the heart. Irisin did not affect the cardiogenic, vasculogenic, or fibrogenic differentiation of ADSCs, suggesting that direct transdifferentiation is not involved. CM from irisin‐treated ADSCs increased the tube formation ability of rCAECs but did not inhibit cardiomyocyte apoptosis in vitro. RNAseq screening followed by quantitative PCR/Western blotting demonstrated that irisin significantly induces the expression of ANGPTL4 in ADSCs. ANGPTL4 is a proangiogenic factor and has been shown to improve the cerebral vasculature network after brain injury.^[^ ^40^ ^]^ ANGPTL4 promotes angiogenesis by increasing nitric oxide production via the JAK/STAT3‐mediated upregulation of inducible nitric oxide synthase expression.^[^ ^41^ ^]^ In the present study, gain‐ and loss‐of‐function studies revealed that ANGPTL4 contributes to the proangiogenic effect of irisin‐treated ADSCs.

Finally, we identified integrin *α*V/*β*5 as the receptor mediating the actions of irisin on ADSCs. One previous study demonstrated that irisin enters mouse alveolar cells via endocytosis and directly targets mitochondria.^[^ ^23^ ^]^ However, we demonstrated that endocytosis is not involved in irisin downstream signaling in ADSCs. Kim et al. first identified integrin *α*V/*β*5 as the irisin receptor in mouse osteocytes and fat tissues.^[^ ^11^ ^]^ Oguri et al. recently reported that both the *α*V/*β*1 and *α*V/*β*5 integrins mediate the activation of integrin‐FAK signaling in response to irisin in a subset of mouse adipocyte progenitor cells.^[^ ^24^ ^]^ Integrins have been shown to promote the homing of several stem/progenitor cells, such as hematopoietic stem cells,^[^ ^42^ ^]^ endothelial progenitor cells,^[^ ^43^ ^]^ and MSCs.^[^ ^44^ ^]^ Leptin increases integrin *α*V/*β*5 expression, which augments the homing of intravenously injected endothelial progenitor cells to the site of vascular injury.^[^ ^45^ ^]^ In the present study, we found that integrins *α*V and *β*5 were not the most highly expressed integrins in ADSCs, as evidenced by the expression patterns of all *α* and *β* integrins in the RNAseq analysis. However, we revealed that integrin *α*V/*β*5, but not *α*V/*β*1, is required for the irisin‐induced upregulation of key downstream molecules in both rADSCs and mADSCs. Irisin lost its ability to activate ERK1/2 when integrin *α*V/*β*5 was genetically blocked. These results support integrin *α*V/*β*5 as the specific receptor of irisin in MSCs.

A recent study by Deng et al. reported that hypoxic exposure decreases the protein expression of FNDC5 in bone marrow‐derived MSCs (BM‐MSCs). BM‐MSCs with FNDC5 overexpression improved the survival of transplanted BM‐MSCs, which significantly reduced fibrosis and alleviated injured heart function in a mouse MI model.^[^ ^46^ ^]^ The final outcome, that is, enhanced cardioprotective ability of FNDC5/irisin pretreated MSCs is the same, demonstrating the reproducibility of our experimental finding. However, several novel aspects clearly distinguish our current study with the previous report. First, and most significantly, the delivery routes of cell therapy are different. We intravenously injected MSCs and demonstrated that irisin increases their homing to the ischemic heart. The previous study directly injected MSCs into the infarcted area. Because intravenous delivery is a noninvasive method that allows multiple injections, it has greater clinical application than intramyocardial injection. Second, we identified for the first time that integrin *α*V/*β*5 is the irisin receptor in ADSCs. Thirdly, we uncovered novel mechanisms mediating the effect of irisin on ADSCs. Specifically, the previous study reported that irisin or FNDC5 overexpression alleviated hypoxic stress‐induced BM‐MSCs apoptosis and cell death. At a cellular level, we demonstrated for the first time that in addition to reduce ADSCs apoptosis (same as previously reported), irisin enhanced the proangiogenic effect of ADSCs. At molecular level, we utilized unbiased approach and demonstrated for the first time that increased CSF2RB, ANGPTL4, and SOD2 expression by irisin is responsible for the increased cardiac homing and cardioprotective ability of ADSCs. Finally, we identified ERK1/2 as the upstream molecule upregulating CSF2RB, SOD2, and ANGPTL4 expression. To our knowledge, this molecular signaling system has never been previously reported in any stem cell–related cardioprotection.

## 4 Conclusion

In conclusion, we identified irisin as a novel myokine that promotes the cardiac engraftment and cardioprotection of intravenously delivered ADSCs via three major mechanisms. First, irisin directs the cardiac homing of ADSCs via integrin *α*V/*β*5‐ERK1/2 activation, followed by the upregulation of CSF2RB expression in response to the CSF2 gradient. Second, irisin promotes ADSC survival by upregulating SOD2 expression. Third, irisin enhances the paracrine function of ADSCs by increasing ANGPTL4 expression and release. Multiple intravenous deliveries of irisin‐pretreated MSCs may be a useful, noninvasive cellular therapeutic strategy for patients with ischemic heart diseases.

## 5 Experimental Section

Experimental details are provided in Supporting Information.

### Study Design

The main goal of this study is to determine whether irisin, an exercise‐related myokine, facilitates the cardiac engraftment and repair of intravenously injected ADSCs in hearts after MI/R. The experiments were carried out in both rats and mice with long‐term MI/R injury, which effectively recapitulates the human disease process. MI/R‐induced animals with an LVEF > 45% at 1 day after the operation were excluded from the study. At least 20 mice and 10 rats were included in the experimental groups to identify alterations in cardiac function. Mice and rats were randomly assigned to treatment groups. Both male and female rats were used to study post‐MI/R cardiac function and fibrosis. To evaluate the organ distribution of intravenously delivered ADSCs, ADSCs was tracked by either lipophilic dye CM‐DiI staining or genetic GFP and tdTomato labeling at two different time points. To identify the downstream molecular mechanisms of irisin, nonbiased RNA‐seq analysis was first performed and then, additional studies were performed. To determine the role of the CSF2‐CSF2RB axis in the irisin‐enhanced cardiac homing of ADSCs, we knocked down CSF2 in heart tissue and knocked down CSF2RB in ADSCs. The chemicals, molecular reagents, and chemically synthesized miRNAs of the highest‐grade purity were purchased from reputable vendors. All sample measurements were performed in a blinded manner.

### Statistical Analysis

All values were presented as the mean±SEM of *n* independent experiments. The results were analyzed by an unpaired, 2‐tailed Student's *t* test (2 groups) or ANOVA (≥3 groups) followed by Bonferroni correction if needed. All statistical tests were performed with GraphPad Prism software version 8.0, and *p* <0.05 was considered significant.

## Conflict of Interest

The authors declare no conflict of interest.

## Supporting information

Supporting Information

## Data Availability

The data that support the findings of this study are available from the corresponding author upon reasonable request.

## References

[1] S. E. Epstein , D. Luger , M. J. Lipinski , Circ. Res. 2017, 121, 1044. 29025759 10.1161/CIRCRESAHA.117.311925

[2] J. D. Richardson , P. J. Psaltis , L. Frost , S. Paton , A. Carbone , A. G. Bertaso , A. J. Nelson , D. T. Wong , M. I. Worthley , S. Gronthos , A. C. Zannettino , S. G. Worthley , Cytotherapy 2014, 16, 460. 24113430 10.1016/j.jcyt.2013.07.016

[3] Y. Guo , M. Wysoczynski , Y. Nong , A. Tomlin , X. Zhu , A. M. Gumpert , M. Nasr , S. Muthusamy , H. Li , M. Book , A. Khan , K. U. Hong , Q. Li , R. Bolli , Basic Res. Cardiol. 2017, 112, 18. 28210871 10.1007/s00395-017-0606-5 PMC5655998

[4] M. Wysoczynski , A. Khan , R. Bolli , Circ. Res. 2018, 123, 138. 29976684 10.1161/CIRCRESAHA.118.313251 PMC6050028

[5] A. Pandey , S. Garg , M. Khunger , D. Darden , C. Ayers , D. J. Kumbhani , H. G. Mayo , J. A. de Lemos , J. D. Berry , Circulation 2015, 132, 1786. 26438781 10.1161/CIRCULATIONAHA.115.015853

[6] U. Corrà , M. F. Piepoli , F. Carré , P. Heuschmann , U. Hoffmann , M. Verschuren , J. Halcox , P. Giannuzzi , H. Saner , D. Wood , M. F. Piepoli , U. Corrà , W. Benzer , B. Bjarnason‐Wehrens , P. Dendale , D. Gaita , H. McGee , M. Mendes , J. Niebauer , A. D. Zwisler , J. P. Schmid , Eur. Heart J. 2010, 31, 1967. 20643803 10.1093/eurheartj/ehq236

[7] R. Recchioni , F. Marcheselli , R. Antonicelli , R. Lazzarini , E. Mensà , R. Testa , A. D. Procopio , F. Olivieri , Mechanisms of ageing and development 2016, 159, 71. 27015708 10.1016/j.mad.2016.03.008

[8] E. M. Van Craenenbroeck , V. Y. Hoymans , P. J. Beckers , N. M. Possemiers , K. Wuyts , B. P. Paelinck , C. J. Vrints , V. M. Conraads , Basic Res. Cardiol. 2010, 105, 665. 20508941 10.1007/s00395-010-0105-4

[9] J. Rehman , J. Li , L. Parvathaneni , G. Karlsson , V. R. Panchal , C. J. Temm , J. Mahenthiran , K. L. March , J. Am. Coll Cardiol. 2004, 43, 2314. 15193699 10.1016/j.jacc.2004.02.049

[10] M. D. Boppart , M. De Lisio , S. Witkowski , Prog. Mol. Biol. Transl. Sci. 2015, 135, 423. 26477925 10.1016/bs.pmbts.2015.07.005

[11] H. Kim , C. D. Wrann , M. Jedrychowski , S. Vidoni , Y. Kitase , K. Nagano , C. Zhou , J. Chou , V. A. Parkman , S. J. Novick , T. S. Strutzenberg , B. D. Pascal , P. T. Le , D. J. Brooks , A. M. Roche , K. K. Gerber , L. Mattheis , W. Chen , H. Tu , M. L. Bouxsein , P. R. Griffin , R. Baron , C. J. Rosen , L. F. Bonewald , B. M. Spiegelman , Cell 2018, 175, 1756. 30550785 10.1016/j.cell.2018.10.025 PMC6298040

[12] P. Boström , J. Wu , M. P. Jedrychowski , A. Korde , L. Ye , J. C. Lo , K. A. Rasbach , E. A. Boström , J. H. Choi , J. Z. Long , S. Kajimura , M. C. Zingaretti , B. F. Vind , H. Tu , S. Cinti , K. Højlund , S. P. Gygi , B. M. Spiegelman , Nature 2012, 481, 463. 22237023 10.1038/nature10777 PMC3522098

[13] M. P. Jedrychowski , C. D. Wrann , J. A. Paulo , K. K. Gerber , J. Szpyt , M. M. Robinson , K. S. Nair , S. P. Gygi , B. M. Spiegelman , Cell Metab. 2015, 22, 734. 26278051 10.1016/j.cmet.2015.08.001 PMC4802359

[14] X. Zhou , M. Xu , J. L. Bryant , J. Ma , X. Xu , Cell Biosci. 2019, 9, 32. 30984367 10.1186/s13578-019-0294-y PMC6446275

[15] W. Yan , Y. Guo , L. Tao , W. B. Lau , L. Gan , Z. Yan , R. Guo , E. Gao , G. W. Wong , W. L. Koch , Y. Wang , X. L. Ma , Circulation 2017, 136, 2162. 28978553 10.1161/CIRCULATIONAHA.117.029557 PMC5705403

[16] W. Yan , C. Lin , Y. Guo , Y. Chen , Y. Du , W. B. Lau , Y. Xia , F. Zhang , R. Su , E. Gao , Y. Wang , C. Li , R. Liu , X. L. Ma , L. Tao , Circ. Res. 2020, 126, 857. 32079489 10.1161/CIRCRESAHA.119.315806

[17] Y. Wu , R. C. Zhao , Stem Cell Rev. Rep. 2012, 8, 243. 21706142 10.1007/s12015-011-9293-z

[18] A. Anzai , J. L. Choi , S. He , A. M. Fenn , M. Nairz , S. Rattik , C. S. McAlpine , J. E. Mindur , C. T. Chan , Y. Iwamoto , B. Tricot , G. R. Wojtkiewicz , R. Weissleder , P. Libby , M. Nahrendorf , J. R. Stone , B. Becher , F. K. Swirski , J. Exp. Med. 2017, 214, 3293. 28978634 10.1084/jem.20170689 PMC5679174

[19] M. Horckmans , M. Bianchini , D. Santovito , R. T. A. Megens , J. Y. Springael , I. Negri , M. Vacca , M. Di Eusanio , A. Moschetta , C. Weber , J. Duchene , S. Steffens , Circulation 2018, 137, 948. 29167227 10.1161/CIRCULATIONAHA.117.028833

[20] D. J. Li , Y. H. Li , H. B. Yuan , L. F. Qu , P. Wang , Metabol.: Clin. Exp. 2017, 68, 31. 10.1016/j.metabol.2016.12.003 28183451

[21] Y. D. Jung , S. K. Park , D. Kang , S. Hwang , M. H. Kang , S. W. Hong , J. H. Moon , J. S. Shin , D. H. Jin , D. You , J. Y. Lee , Y. Y. Park , J. J. Hwang , C. S. Kim , N. Suh , Redox Biol. 2020, 37, 101716. 32961441 10.1016/j.redox.2020.101716 PMC7509080

[22] D. I. Cho , H. J. Kang , J. H. Jeon , G. H. Eom , H. H. Cho , M. R. Kim , M. Cho , H. Y. Jeong , H. C. Cho , M. H. Hong , Y. S. Kim , Y. Ahn , JCI Insight 2019, 4, 10.1172/jci.insight.125437 PMC6777833 31434807

[23] K. Chen , Z. Xu , Y. Liu , Z. Wang , Y. Li , X. Xu , C. Chen , T. Xia , Q. Liao , Y. Yao , C. Zeng , D. He , Y. Yang , T. Tan , J. Yi , J. Zhou , H. Zhu , J. Ma , C. Zeng , Sci. Transl. Med. 2017, 9, eaao6298 29187642 10.1126/scitranslmed.aao6298 PMC5969805

[24] Y. Oguri , K. Shinoda , H. Kim , D. L. Alba , W. R. Bolus , Q. Wang , Z. Brown , R. N. Pradhan , K. Tajima , T. Yoneshiro , K. Ikeda , Y. Chen , R. T. Cheang , K. Tsujino , C. R. Kim , V. J. Greiner , R. Datta , C. D. Yang , K. Atabai , M. T. McManus , S. K. Koliwad , B. M. Spiegelman , S. Kajimura , Cell 2020, 182, 563. 32615086 10.1016/j.cell.2020.06.021 PMC7415677

[25] Z. Sun , Y. Xie , R. J. Lee , Y. Chen , Q. Jin , Q. Lv , J. Wang , Y. Yang , Y. Li , Y. Cai , R. Wang , Z. Han , L. Zhang , M. Xie , Theranostics 2020, 10, 4967. 32308762 10.7150/thno.43233 PMC7163444

[26] J. Wang , Z. Chen , Q. Dai , J. Zhao , Z. Wei , J. Hu , X. Sun , J. Xie , B. Xu , Basic Res. Cardiol. 2020, 115, 40. 32451935 10.1007/s00395-020-0800-8

[27] T. L. Laundos , F. Vasques‐Nóvoa , R. N. Gomes , V. Sampaio‐Pinto , P. Cruz , H. Cruz , J. M. Santos , R. N. Barcia , P. Pinto‐do‐Ó , D. S. Nascimento , Front. Cell Dev. Biol. 2021, 9, 624601. 33614654 10.3389/fcell.2021.624601 PMC7890004

[28] J. Bartolucci , F. J. Verdugo , P. L. González , R. E. Larrea , E. Abarzua , C. Goset , P. Rojo , I. Palma , R. Lamich , P. A. Pedreros , G. Valdivia , V. M. Lopez , C. Nazzal , F. Alcayaga‐Miranda , J. Cuenca , M. J. Brobeck , A. N. Patel , F. E. Figueroa , M. Khoury , Circ. Res. 2017, 121, 1192. 28974553 10.1161/CIRCRESAHA.117.310712 PMC6372053

[29] D. Luger , M. J. Lipinski , P. C. Westman , D. K. Glover , J. Dimastromatteo , J. C. Frias , M. T. Albelda , S. Sikora , A. Kharazi , G. Vertelov , R. Waksman , S. E. Epstein , Circ. Res. 2017, 120, 1598. 28232595 10.1161/CIRCRESAHA.117.310599

[30] R. H. Lee , A. A. Pulin , M. J. Seo , D. J. Kota , J. Ylostalo , B. L. Larson , L. Semprun‐Prieto , P. Delafontaine , D. J. Prockop , Cell Stem Cell 2009, 5, 54. 19570514 10.1016/j.stem.2009.05.003 PMC4154377

[31] W. Xiao , T. I. P. Green , X. Liang , R. C. Delint , G. Perry , M. S. Roberts , K. L.e Vay , C. R. Back , R. Ascione , H. Wang , P. R. Race , A. W. Perriman , Chem. Sci. 2019, 10, 7610. 31588312 10.1039/c9sc02650a PMC6764276

[32] A. D. Anastasilakis , D. Koulaxis , N. Kefala , S. A. Polyzos , J. Upadhyay , E. Pagkalidou , F. Economou , C. D. Anastasilakis , C. S. Mantzoros , Metabol. Clin. Exp. 2017, 73, 1. 10.1016/j.metabol.2017.05.002 28732565

[33] T. H. Efe , B. Açar , A. G. Ertem , K. G. Yayla , E. Algül , Ç. Yayla , S. Ünal , M. Bilgin , T. Çimen , Ö. Kirbaş , E. Yeter , Korean Circ. J. 2017, 47, 44. 28154590 10.4070/kcj.2016.0079 PMC5287186

[34] Q. Liao , S. Qu , L. X. Tang , L. P. Li , D. F. He , C. Y. Zeng , W. E. Wang , Acta Pharmacol. Sin. 2019, 40, 1314. 31061533 10.1038/s41401-019-0230-z PMC6786355

[35] T. Xin , C. Lu , Aging 2020, 12, 4474. 32155590 10.18632/aging.102899 PMC7093202

[36] H. Wang , Y. T. Zhao , S. Zhang , P. M. Dubielecka , J. Du , N. Yano , Y. E. Chin , S. Zhuang , G. Qin , T. C. Zhao , J. Cell. Physiol. 2017, 232, 3775. 28181692 10.1002/jcp.25857 PMC5550372

[37] S. R. Park , A. Cho , J. W. Kim , H. Y. Lee , I. S. Hong , Mol. Ther. 2019, 27, 1087. 30962162 10.1016/j.ymthe.2019.03.010 PMC6554530

[38] L. Zhang , G. Liu , K. Lv , J. Xin , Y. Wang , J. Zhao , W. Hu , C. Xiao , K. Zhu , L. Zhu , J. Nan , Y. Feng , H. Zhu , W. Chen , W. Zhu , J. Zhang , J. Wang , B. Wang , X. Hu , Adv. Sci. 2021, 8, 2003348. 10.1002/advs.202003348 PMC7856906 33552872

[39] X. Zhang , C. Hu , C. Y. Kong , P. Song , H. M. Wu , S. C. Xu , Y. P. Yuan , W. Deng , Z. G. Ma , Q. Z. Tang , Cell Death Differ. 2020, 27, 540. 31209361 10.1038/s41418-019-0372-z PMC7206111

[40] C. Bouleti , T. Mathivet , B. Coqueran , J. M. Serfaty , M. Lesage , E. Berland , C. Ardidie‐Robouant , G. Kauffenstein , D. Henrion , B. Lapergue , M. Mazighi , C. Duyckaerts , G. Thurston , D. M. Valenzuela , A. J. Murphy , G. D. Yancopoulos , C. Monnot , I. Margaill , S. Germain , Eur. Heart J. 2013, 34, 3657. 23674618 10.1093/eurheartj/eht153

[41] H. C. Chong , J. S. Chan , C. Q. Goh , N. V. Gounko , B. Luo , X. Wang , S. Foo , M. T. Wong , C. Choong , S. Kersten , N. S. Tan , Mol. Ther. 2014, 22, 1593. 24903577 10.1038/mt.2014.102 PMC4435481

[42] E. Taniguchi Ishikawa , K. H. Chang , R. Nayak , H. A. Olsson , A. M. Ficker , S. K. Dunn , M. N. Madhu , A. Sengupta , J. A. Whitsett , H. L. Grimes , J. A. Cancelas , Nat. Commun. 2013, 4, 1660. 23552075 10.1038/ncomms2645 PMC3627399

[43] G. Carmona , E. Chavakis , U. Koehl , A. M. Zeiher , S. Dimmeler , Blood 2008, 111, 2640. 18032709 10.1182/blood-2007-04-086231

[44] J. E. Ip , Y. Wu , J. Huang , L. Zhang , R. E. Pratt , V. J. Dzau , Mol. Biol. Cell 2007, 18, 2873. 17507648 10.1091/mbc.E07-02-0166 PMC1949353

[45] M. R. Schroeter , M. Leifheit , P. Sudholt , N. M. Heida , C. Dellas , I. Rohm , F. Alves , M. Zientkowska , S. Rafail , M. Puls , G. Hasenfuss , S. Konstantinides , K. Schäfer , Circ. Res. 2008, 103, 536. 18658052 10.1161/CIRCRESAHA.107.169375

[46] J. Deng , N. Zhang , Y. Wang , C. Yang , Y. Wang , C. Xin , J. Zhao , Z. Jin , F. Cao , Z. Zhang , Stem Cell Res. Ther. 2020, 11, 228. 32522253 10.1186/s13287-020-01746-z PMC7288492

