# Supplementary material for: Irisin Promotes Cardiac Homing of Intravenously Delivered MSCs and Protects against Ischemic Heart Injury
Source: Adv Sci (Weinh). 2022 Jan 17;9(7):2103697. doi: 10.1002/advs.202103697 (PMC8895138; doi:10.1002/advs.202103697)
Supplement: Supplementary file 1 — Supporting Information [file ADVS-9-2103697-s001.pdf]

ADVANCED SCIENCE
Open Access

# Supporting Information

for *Adv. Sci.,* DOI: 10.1002/advs.202103697

# Irisin promotes cardiac homing of intravenously delivered MSCs and protects against ischemic heart injury

*Wenjun Yan *, Youhu Chen, Yongzhen Guo, Yunlong Xia, Congye Li, Yunhui Du, Chen Lin, Xiaoming Xu, Tingting Qi, Miaomiao Fan, Fuyang Zhang, Guangyu Hu, Erhe Gao, Rui Liu, Chunxu Hai, Ling Tao**

# Experimental methods

### Animals

Animal experiments were approved by the Animal Care and Use Committee of the Fourth Military Medical University. All experiments were performed in strict accordance with the National Institutes of Health (NIH) guidelines on the Use of Laboratory Animals (NIH publication no. 85-23, revised 2011). EGFP-expressing transgenic mice on a C57BL/6J background were purchased from The Jackson Laboratory (Stock 003291) [1]. TdTomato-expressing transgenic rats on a Sprague-Dawley background were purchased from Shanghai Model Organisms (NR-KI-190007). Adult (10–12 weeks) C57BL/6J mice, adult (10–12 weeks) Sprague-Dawley rats, and Sprague-Dawley pups (1–2 days) were purchased from the Laboratory Animal Center of the Fourth Military Medical University.

### Materials

Recombinant irisin (human, mouse, rat) in monomer was purchased from Aviscera Bioscience (00170-01-10). Recombinant mouse ANGPTL4 was purchased from MedChemExpress (HY-P7508). The lipophilic dye collagenase type I (17018029), collagenase type II (17101015), and CM-DiI (C7001) were purchased from Thermo Fisher Scientific. The cell plasma membrane staining kit with DiO was purchased from Beyotime Biotechnology (C1993S). The red blood cell lysis buffer was purchased from TianGen BioTech (RT122-02).

### Animal study protocol

We used a modified surgical model of MI/R for more complete killing of the ischemic zone and greater reproducibility. Mice or rats were anesthetized by inhalation of 1–2% isoflurane, and left anterior descending coronary artery ligation surgery was performed as described previously [2]. After 120 minutes of MI, the slipknot was

released, and the myocardium was reperfused. One day after MI/R, echocardiography was performed. MI/R animals with an LVEF>45% were excluded from the study. Each mouse was injected via the vena angularis with $5\times10^5$ ADSCs suspended in 100 μL of PBS (containing 0.2 mM EDTA, pH=7.3) at 1 day, 8 days, 15 days, 22 days, and 29 days after the MI/R operation. Each rat was injected via the vena sublingualis with $5\times10^6$ ADSCs suspended in 300 μL of PBS (containing 0.2 mM EDTA, pH=7.3) at 1 day, 8 days, 15 days, 22 days, and 29 days after the MI/R operation. MI/R control animals received only a saline injection. Sham control animals were subjected to all surgical procedures except for coronary artery ligation.

For the MI/R mouse model, adult male C57BL/6J mice were randomly assigned to the following groups: Sham, MI/R+vehicle, MI/R+ADSC-vehicle (CM-DiI-labeled ADSCs treated with vehicle), and MI/R+ADSC-irisin (CM-DiI-labeled ADSCs treated with 100 ng/mL irisin for 24 hours and then washed with PBS). For CSF2/CSF2RB loss-of-function studies, one group of adult male mice were intramyocardially injected with AAV9-control ($CSF2^{WT}$) or AAV9-CSF2-shRNA ($CSF2^{KD}$) one month before they were divided into the following groups: $CSF2^{WT}$ +sham; $CSF2^{WT}$ +MI/R; $CSF2^{WT}$ +MI/R+ADSC-irisin; $CSF2^{KD}$ +sham; $CSF2^{KD}$ +MI/R; and $CSF2^{KD}$ +MI/R+ADSC-irisin. Another group of mice were randomly subjected to the following five groups: MI/R+ADSC-vehicle (ADSCs were treated with vehicle); MI/R+ADSC-irisin-$CSF2RB^{WT}$ (ADSCs were treated with irisin after they were transfected with scRNA); MI/R+ADSC-irisin-$CSF2RB^{KD}$ (ADSCs were treated with irisin after they were transfected with siRNA against CSF2RB); MI/R+ADSC-irisin+IgG (IgG was intravenously injected with ADSC-irisin); and MI/R+ADSC-irisin+CSF2-BA (the blocking antibody of CSF2 was intravenously injected with ADSC-irisin).

For the MI/R rat model, adult male and female Sprague-Dawley rats were randomly assigned to the following groups: Sham; MI/R+vehicle; MI/R+rADSC-vehicle; and MI/R+rADSC-irisin.

**ADSC preparation**

ADSCs were isolated from adult male C57BL/6J mice and adult male Sprague-Dawley rats as we previously described [3]. Briefly, inguinal subcutaneous adipose tissue was removed under anesthesia. The adipose tissue was rinsed several times with phosphate buffered saline (PBS). Blood vessels were excised under a dissection microscope. The remaining adipose tissue was cut into fine pieces, digested with 0.1% collagenase type I at 37°C for 60 minutes, and centrifuged at 600 g for 10 minutes. After red blood cell lysis with 1× lysis buffer, the cells were cultured in a 1:1 mixture of Dulbecco’s modified Eagle’s medium (DMEM) and F12 medium containing 10% fetal bovine serum (FBS) and penicillin-streptomycin. Six hours after cell plating, the medium was changed to remove nonadherent cells. Adherent cells were cultured in DMEM-F12-10% FBS and split several times for expansion. Cells from passages 2–3 were used in all experiments.

**Adenovirus construction and transfection**

The recombinant FNDC5 adenovirus was constructed by Likely Biotechnology (Beijing, China). Cells were infected with the FNDC5 adenovirus or adenovirus containing empty plasmids (control) for 24 hours at a multiplicity of infection (MOI) of 20–200. The medium was then replaced with fresh medium, and the cells were cultured for another 24 hours.

**Adeno-associated virus serotype-9 construction and intramyocardial injection**

Adeno-associated virus serotype-9 carrying CSF2 shRNA (AAV9-CSF2-shRNA) or scramble RNA (AAV9-control) was purchased from GenePharma. One month

before the MI/R operation, AAV9-CSF2-shRNA or AAV9-control ($2.6\times10^{10}$ vector genomes per mouse) was intramyocardially injected into the left ventricle free wall at three different sites. The experiments were performed by an investigator who was blinded to the group allocations.

**Small interfering RNA (siRNA)-mediated gene knockdown**

ANGPTL4 siRNA, CSF2RB siRNA, SOD2 siRNA, and their scramble RNAs were purchased from GenePharma. When ADSCs reached 80% confluence, siRNAs were transfected into the cells with RNAiMAX Transfection Reagent (Thermo Fisher Scientific, 13778075) according to the manufacturer's protocol (final siRNA concentration: 100 nM). After 8 hours of incubation (37°C), the transfection reagent-siRNA mixture was replaced with fresh growth medium. Successful knockdown was confirmed by Western blot analysis of the ANGPTL4, CSF2RB, and SOD2 protein levels.

**ADSC labeling**

Cultured ADSCs were labeled with the lipophilic dye CM-DiI (5 µM in DMEM-F12-10% FBS) for 20 minutes immediately before intravenous injection. ADSCs were also stained with cell plasma membrane staining kit with DiO, according to the manufacturer's protocol.

**Flow cytometric analysis of the cardiac homing of ADSCs**

The cardiac homing of ADSCs was evaluated by flow cytometry 1 day after the fifth intravenous injection (30 days after MI/R). Mice were heparinized (10 IU/g body weight), and 5 minutes later, they were anesthetized with an intraperitoneal injection of ketamine/xylazine mixture (100 mg/kg ketamine plus 10 mg/kg xylazine). Single cells were then enzymatically isolated from the hearts in 0.1% collagenase type II (30 minutes at 37°C) as we previously described [4]. A 'myocyte-depleted' cardiac cell

population was prepared by filtering the single cells through a 40-μm mesh and was then stained with a PE/Cy5 anti-mouse/rat CD29 antibody (#102219, Biolegend). A PE/Cy5 Armenian hamster IgG isotype control antibody (#400909, Biolegend) was used as the negative control. The cardiac homing of ADSCs (CM-DiI+/CD29+) was determined with a flow cytometer (Epics MCL, Beckman).

**The *in vivo* competitive homing assay**

The competitive homing assay [5] was performed after a single intravenous injection (1 day after MI/R), and the distribution of ADSCs in organs was evaluated by flow cytometry. Vehicle-treated ADSCs (ADSC-vehicle) were labeled with DiO (a green dye) and irisin-treated ADSCs (ADSC-irisin) were labeled with CM-DiI (a red dye). Equal number of ADSC-vehicle-DiO and ADSC-irisin-DiI were then mixed before they were intravenously injected into the post-MI/R mice. An aliquot of the cell mixture was kept and analyzed by flow cytometry. The mice were sacrificed 1 day after intravenous injection (2 days after MI/R). The hearts, lungs, and spleens were removed from the mice. The hearts were prepared for the 'myocyte-depleted' cardiac cell population. The lungs were minced and then digested in 0.1% collagenase type II (20 minutes at 37°C). Spleens were homogenized into a single-cell suspension using PBS. After red blood cell lysis with 1× lysis buffer, the lung cells and splenocytes were resuspended in PBS. Flow cytometry assays were performed to detect ADSC-vehicle-DiO and ADSC-irisin-DiI in the 'myocyte-depleted' cardiac cell population and single-cell suspensions of lung and spleen.

**Echocardiography**

M-mode images of mice and rats subjected to 1–2% isoflurane anesthesia were obtained via a VisualSonics 770 echocardiography machine (Canada) as previously described [6]. Hearts were viewed along the long and short axes between the two

papillary muscles. The LV end-systolic diameter (LVESD) and LV end-diastolic dimension (LVEDD) were measured. The LVEF was automatically calculated by echocardiography software as follows: EF (%)=100×[($LVEDD^3$-$LVESD^3$)/$LVEDD^3$]. Measurements were performed for at least five separate cardiac cycles per mouse. Echocardiography was performed by a single experienced operator in a blinded fashion.

**Evaluation of angiogenesis**

For the evaluation of angiogenesis, heart sections were deparaffinized and subjected to antigen retrieval in hot citric acid buffer. After cooling, the slides were permeabilized with 0.2% Triton-100 for 15 minutes, blocked with 1% BSA in PBS for 2 hours, and incubated with an anti-CD31 primary antibody at 4°C overnight (#GB13063, Servicebio). A donkey anti-goat antibody conjugated with CY3 (#GB21404, Servicebio) served as the secondary antibody. Nuclei were stained with 4',6-diamidino-2-phenylindole (DAPI, GB1012, Servicebio). Images of immunostained sections were acquired with a Nikon Eclipse C1 microscope and Nikon DS-U3 camera. The myocardial capillary density was quantified by Image-Pro Plus 6.0 software (Media Cybernetics). The representative image for each group was selected based upon the mean value.

**Determination of apoptosis**

ADSC apoptosis was determined by TUNEL staining with a One Step TUNEL Apoptosis Assay Kit (Beyotime, C1090). Images were acquired with a Nikon Eclipse Ni microscope and Nikon DS-Ri2 camera. Apoptosis of cardiomyocytes in heart tissue was determined with a Roche In Situ Cell Death Detection Kit (Sigma, 11767305001 and 11767291910) according to the manufacturer’s protocol. Images were acquired with a Nikon Eclipse C1 microscope and Nikon DS-U3 camera. The index of apoptosis

was determined by the number of TUNEL-positive nuclei/total nuclei. The representative image for each group was selected based on the mean value.

**Masson's trichrome staining**

Hearts were harvested from anesthetized mice and rats and embedded in paraffin. The heart tissue extending from just distal to the coronary ligation point to the apex was separated into different segments at 200 μm intervals. Serial 5-μm-thick sections were obtained from each segment for Masson's trichrome staining according to the manufacturer's protocol (Sigma, HT15). Microscopic images of mouse heart sections were obtained with a 1.25× object lens (Nikon, Japan). Images of rat heart sections were acquired with a Nikon Eclipse C1 microscope and Nikon DS-U3 camera. For quantification, measurements of 5 transverse heart sections were analyzed. Fibrotic size was determined as the average ratio of the fibrotic area to the LV area (fibrotic size %) with Image-Pro Plus 6.0 software (Media Cybernetics).

**CM collection**

We employed a modified method to prepare the CM from ADSCs [3]. CM was generated as follows: after growth to 90% confluence in 6-well dishes, ADSCs were pretreated with 100 ng/mL irisin or vehicle for 1 day. The culture medium was washed and replaced with serum-free DMEM/F12 medium. Twenty-four hours later, the serum-free DMEM/F12 medium was collected and centrifuged at 1000 g for 10 minutes to obtain the supernatant (CM).

***In vitro* cardiomyocyte apoptosis assay**

Both primary NRVCs and iPSC-CMs were used for the *in vitro* cardiomyocyte apoptosis assay. Primary cultures of NRVCs from 1- to 2-day-old Sprague-Dawley pups were prepared as described previously [7]. Human iPSC-CMs were purchased from HELP Therapeutics (#NC20010435). The differentiation and preparation of iPSC-CMs

have been described previously [8]. For the evaluation of cardiomyocyte apoptosis, cultured NRVCs and iPSC-CM were subjected to 6 hours of $H_2O_2$ (200 μM) before treatment with recombinant irisin (100 ng/mL), ANGPTL4 (2 μg/mL), or CM derived from ADSCs.

**Capillary-like tube formation assay**

rCAECs were purchased from Procell (CP-R081, Wuhan, China) and cultured in low-glucose DMEM containing endothelial cell growth supplement (#211-GS, Cell Applications), 10% FBS, and penicillin-streptomycin. For the tube formation assay, Matrigel (BD Biosciences) was added to each well of a 48-well plate and allowed to polymerize at 37°C for 30 minutes. rCAECs were treated with CM derived from ADSCs for 24 hours. Images of the formation of capillary-like structures were obtained by computer-assisted microscopy. The total length per field was calculated from five random fields.

**Immunohistochemistry**

For fixed tissues, wax blocks were cut into 5-μm-thick sections and mounted on glass slides for staining. The slides were deparaffinized and subjected to antigen retrieval in hot citric acid buffer. After cooling, the slides were permeabilized with 0.2% Triton-100 for 15 minutes, blocked with 1% BSA in PBS for 2 hours, and incubated overnight at 4°C with an anti-troponin T mouse monoclonal antibody (Thermo Fisher Scientific, MS-295-P0) (1/1,000). The primary antibody was visualized with a donkey anti-mouse IgG (H+L) secondary antibody conjugated with Alexa Fluor 488 (A77440, Yeasen, China). The frozen sections were fixed in acetone at 4°C for 15 min. The sections were blocked with PBS containing 1% BSA at room temperature. Sections were then incubated overnight at 4°C with an anti-TNNT2 rabbit polyclonal antibody (Affinity Biosciences, DF6261). The primary antibody was visualized with a goat anti-

rabbit IgG (H+L) secondary antibody conjugated with Alexa Fluor 488 (33106ES60, Yeasen, China). Nuclei were stained with DAPI (GB1012, Servicebio). Images were acquired with a Nikon Eclipse C1 microscope and Nikon DS-U3 camera.

**RNA sequencing analysis**

Differential gene expression analysis was performed using RNAseq at Shanghai Biotree Biological Technology [9]. After treatment with vehicle or irisin for 24 hours, total RNA was extracted from ADSCs via an RNeasy Mini Kit (Qiagen, 74106) according to the manufacturer's protocol. RNA purity was assessed using a NanoPhotometer® spectrophotometer (Implen). RNA integrity was assessed using the RNA Nano 6000 Assay Kit on the Bioanalyzer 2100 system (Agilent Technologies).

A total of 1 µg of RNA per sample was used as input material for the RNA sample preparations. Sequencing libraries were generated using the NEBNext® UltraTM RNA Library Prep Kit for Illumina® (NEB, USA) according to the manufacturer's recommendations, and index codes were added to attribute sequences to each sample. Briefly, mRNA was purified from total RNA using poly-T oligo-attached magnetic beads. Fragmentation was carried out using divalent cations under elevated temperature conditions in NEBNext First Strand Synthesis Reaction Buffer (5X). First-strand cDNA was synthesized using random hexamer primers and M-MuLV Reverse Transcriptase (RNase H-). Second-strand cDNA synthesis was subsequently performed using DNA Polymerase I and RNase H. The remaining overhangs were converted into blunt ends via exonuclease/polymerase activities. After adenylation of the 3' ends of the DNA fragments, an NEBNext adaptor with hairpin loop structures was ligated to prepare for hybridization. For the preferential selection of cDNA fragments 250~300 bp in length, the library fragments were purified with the AMPure XP system (Beckman Coulter, Beverly, USA). Then, 3 µL of USER Enzyme (NEB, USA) was incubated with size-

selected, adaptor-ligated cDNA for 15 minutes at 37°C and then at 95°C for 5 min prior to performing PCR. Then, PCR was performed with Phusion High-Fidelity DNA polymerase, universal PCR primers and Index (X) Primer. Finally, the PCR products were purified (AMPure XP system), and library quality was assessed on the Agilent Bioanalyzer 2100 system. The library preparations were sequenced on an Illumina NovaSeq platform, and 150 bp paired-end reads were generated.

Differential expression analysis was performed using the DESeq2 R package (1.16.1). DESeq2 provides statistical routines for determining differential expression among digital gene expression data using a model based on the negative binomial distribution. The resulting P-values were adjusted using Benjamini and Hochberg's approach for controlling the false discovery rate. Genes with an adjusted P-value <0.05 found by DESeq2 were assigned as differentially expressed.

Gene Ontology (GO) enrichment analysis of differentially expressed genes was implemented by the clusterProfiler R package, during which gene length bias was corrected. GO terms with corrected P values less than 0.05 were considered to be significantly enriched by the differentially expressed genes. The KEGG database is a resource for understanding the high-level functions and utilities of biological systems, such as cells, organisms and ecosystems, based on molecular-level information, especially from large-scale molecular datasets generated by genome sequencing and other high-throughput experimental technologies (http://www.genome.jp/kegg/). We used the clusterProfiler R package to assess the statistical enrichment of differentially expressed genes in KEGG pathways.

**Quantitative PCR**

Total RNA was extracted from cells via an RNeasy Mini Kit (Qiagen, 74106) according to the manufacturer's protocol. cDNA was prepared from 1 μg of total RNA

using the SuperScript III First-Strand Synthesis System (Thermo Fisher Scientific, 18080051) according to the manufacturer's protocol. PCR was performed via a 7900HT Fast Real-Time PCR System (Applied Biosystems). Samples were analyzed in triplicate in 10 μL reactions according to the SYBR Green PCR Master Mix protocol (Thermo Fisher Scientific, 4472908). Primers were purchased from TsingKe (listed in Table IV in the Data Supplement). β-actin served as the housekeeping gene. Data were normalized via the standard comparative CT method.

The thermal cycling conditions were as follows: denaturation at 95°C for 5 minutes, followed by 40 cycles of 10 s at 95°C, 20 s at 55°C, and 20 s at 72°C.

**Western blot analysis**

Total proteins were isolated from cells or heart tissues with 1× lysis buffer (CST #9803) supplemented with a protease inhibitor cocktail (Thermo Fisher Scientific, 78438). A total of 30–70 μg of protein per sample was separated via gel electrophoresis, transferred to a polyvinylidene fluoride membrane, and blocked with 5% milk for 1 hour. The membrane was incubated overnight with primary antibodies at 4°C. After incubation with a secondary HRP-conjugated anti-mouse antibody (Abbkine, A21010, 1/10,000) or anti-rabbit antibody (Abbkine, A21020, 1/10,000) at room temperature for 2 hours, the membranes were exposed to enhanced chemiluminescent (ECL) substrate (Thermo Fisher Scientific, 34096). The Western blot results were quantified by densitometry (Image Lab).

The following primary antibodies were used in this study: anti-FNDC5/irisin antibody (SinoBiogical, 106683-T08); anti-CSF2RB antibody (Santa Cruz, sc-393281); anti-CSF2 antibody (Santa Cruz, sc-32753); anti-cleaved caspase-3 antibody (CST #9661); anti-caspase-3 antibody (CST #9662); anti-phospho-ERK1/2 antibody (CST #9101); anti-ERK1/2 antibody (CST #4695); anti-phospho-Akt$^{Ser473}$ antibody (CST

#4060); anti-AKT antibody (CST #4691); anti-ANGPTL4 antibody (Abcam, ab196746); anti-MCP1 antibody (Affinity Biosciences, DF7577); anti-IL1β antibody (Affinity Biosciences, AF5103); anti-LCN2 antibody (Affinity Biosciences, DF6816); anti-SOD2 antibody (Santa Cruz, sc-133134); anti-TFPI2 antibody (Abcam, ab186747); anti-integrin αV antibody (CST #4711); anti-integrin β1 antibody (CST #34971); anti-integrin β5 antibody (CST #3629); and anti-GAPDH antibody (ProteinTech, 10494-1-AP).

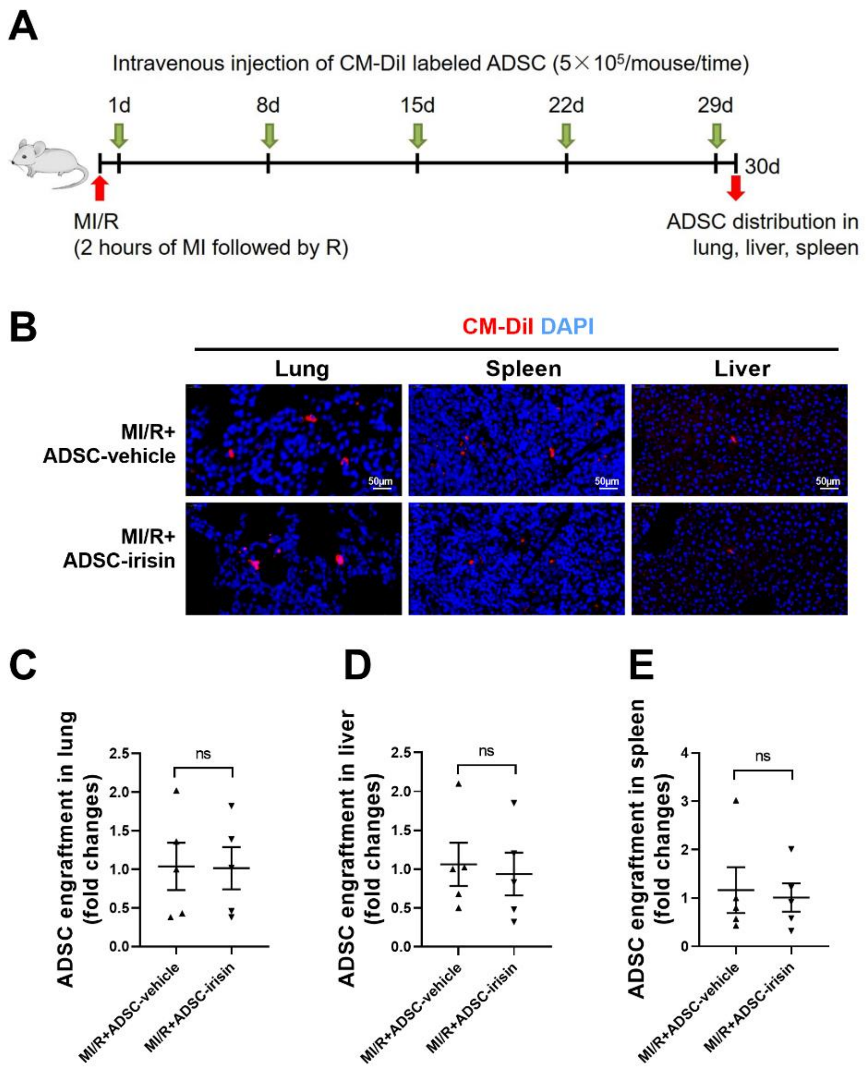


**Figure S1. Irisin does not significantly alter the lung, spleen, or liver distribution of intravenously injected ADSCs.**

A. Experimental scheme and timeline for *in vivo* mouse studies. At 1, 8, 15, 22, and 29 days after myocardial ischemia/reperfusion (MI/R) operation, $5\times10^5$ CM-DiI+ ADSCs were infused intravenously via the vena angularis. ADSC distributions in the lung, liver, and spleen were determined 30 days after MI/R.

B. Representative images of CM-DiI-labeled ADSCs in the lung, liver, and spleen 30 days after MI/R. Engrafted ADSCs are CM-DiI positive (red).

C-E. Quantification of CM-DiI-labeled ADSCs in the lung, liver, and spleen (n=5 mice per group).

The data were analyzed by unpaired, 2-tailed Student’s t test. ADSC-vehicle, ADSCs treated with vehicle; ADSC-irisin, ADSCs treated with irisin (100ng/mL) for 2 days. ns, not significant.

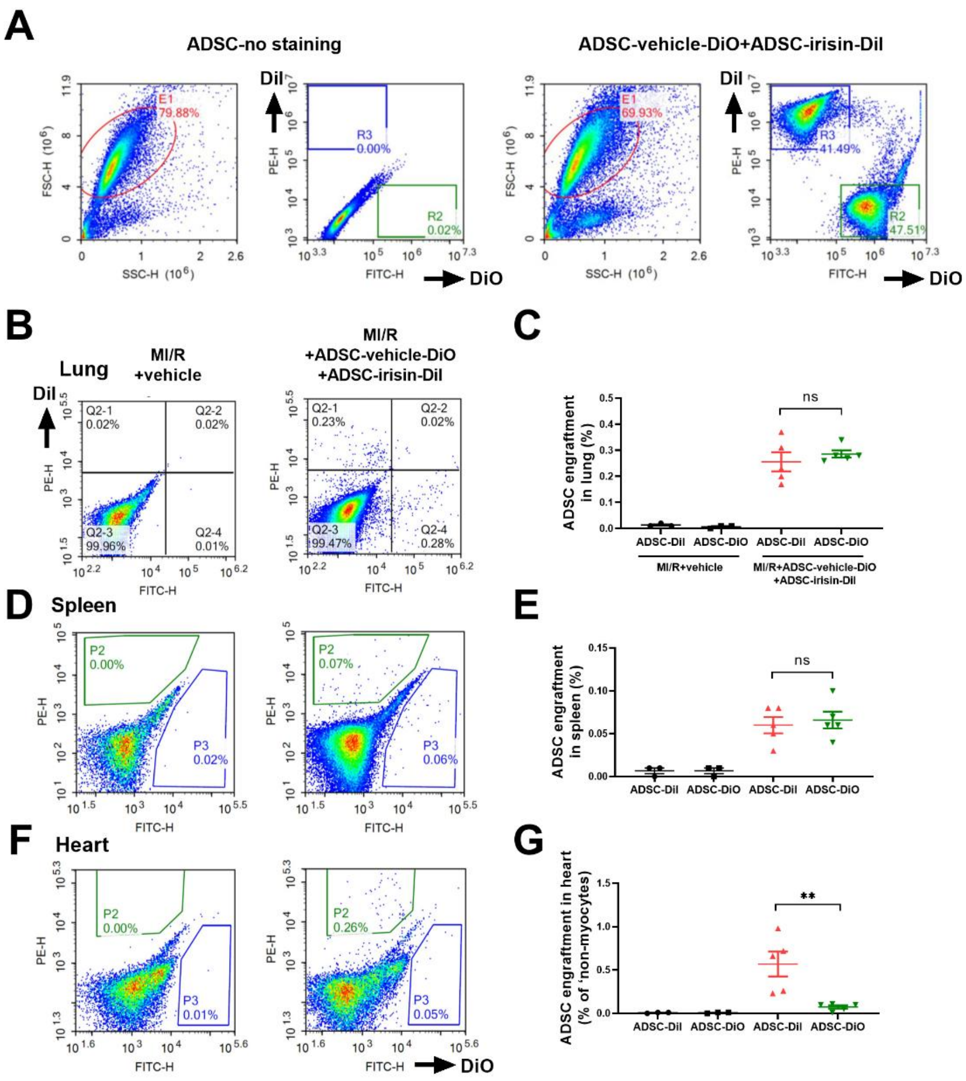


**Figure S2. Irisin increases the cardiac engraftment of intravenously delivered ADSCs.**

A. Flow cytometric analysis of a cell mixture of ADSC-vehicle with DiO staining (ADSC-vehicle-DiO) and ADSC-irisin with CM-DiI staining (ADSC-irisin-DiI).

B-G. Flow cytometric analysis of ADSC-vehicle-DiO and ADSC-irisin-DiI in lungs (B and C), spleens (D and E), and hearts (F and G) 1 day after intravenous injection (2 days after MI/R). n=3-5 mice per group.

The data were analyzed by one-way ANOVA, followed by a Bonferroni *post hoc* test. **P<0.01; ns, not significant.

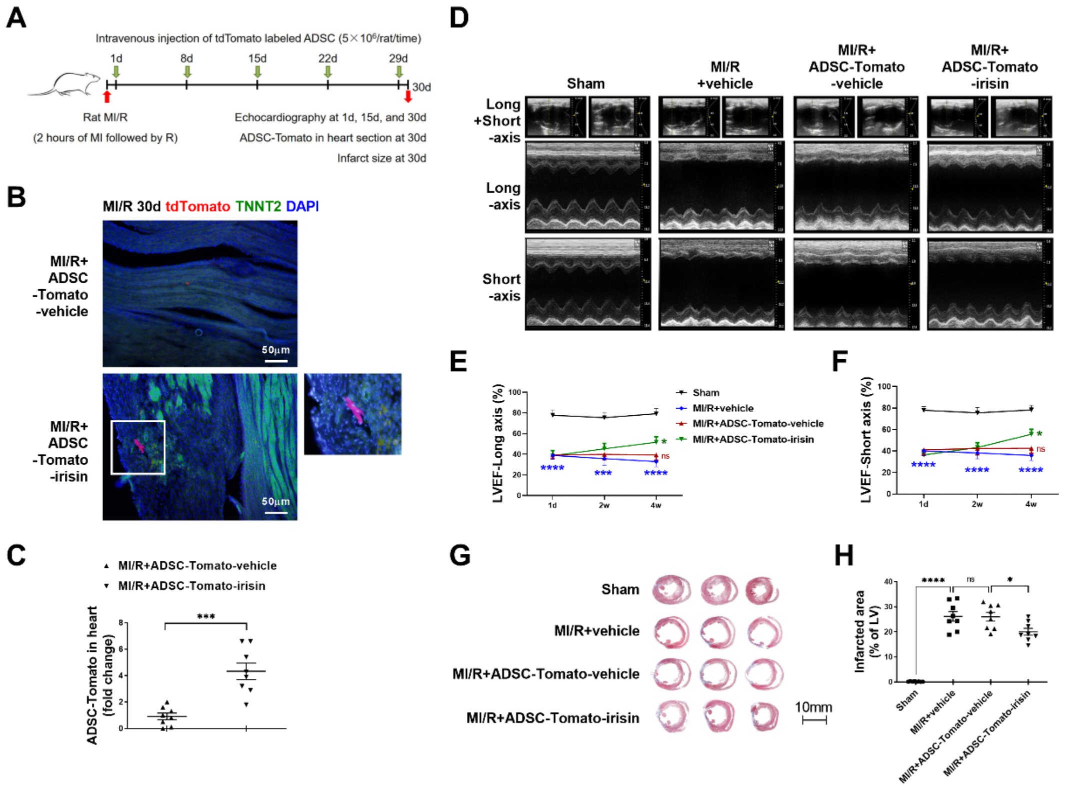


**Figure S3. Irisin enhances the post-MI/R cardioprotection of intravenously delivered ADSCs in rats.**

A. Experimental scheme and timeline for *in vivo* rat studies. At 1, 8, 15, 22, and 29 days after myocardial ischemia/reperfusion (MI/R) operation, $5\times10^6$ tdTomato+ ADSCs were infused intravenously (i.v.) injected via the vena sublingualis.

B. Representative images of ADSC-Tomato in the heart 2 days after MI/R. Heart tissue was immunostained for TNNT2 (green) and DAPI (blue). Engrafted ADSCs are tdTomato positive (red). C. Quantification of ADSCs in the peri-infarct area was determined by the number of ADSC-Tomato in a representative section (n=8 mice per group).

D. Representative long-axis and short-axis M-mode echocardiographic images of rats 30 days after MI/R.

E. Left ventricular ejection fraction (LVEF) evaluated by long-axis M-mode echocardiography. F. LVEF evaluated by short-axis M-mode echocardiography. n=6–10. The data were analyzed by 1-way ANOVA, followed by a Bonferroni post hoc test. ****$P<0.0001$ vs. the Sham group, $^{\#}P<0.05$ vs. the MI/R+ADSC-Tomato-vehicle group.

G. Representative Masson trichrome staining. H. Quantification of the fibrotic area 30 days after MI/R (n=8 rats). The data were analyzed by Kruskal–Wallis test, followed by Dunn *post hoc* tests.

ADSC-Tomato, ADSCs isolated from transgenic rats expressing tdTomato. ADSC-Tomato-vehicle, ADSC-Tomato treated with vehicle; ADSC-Tomato-irisin, ADSC-Tomato treated with irisin (100ng/mL) for 2 days. ns, not significant.

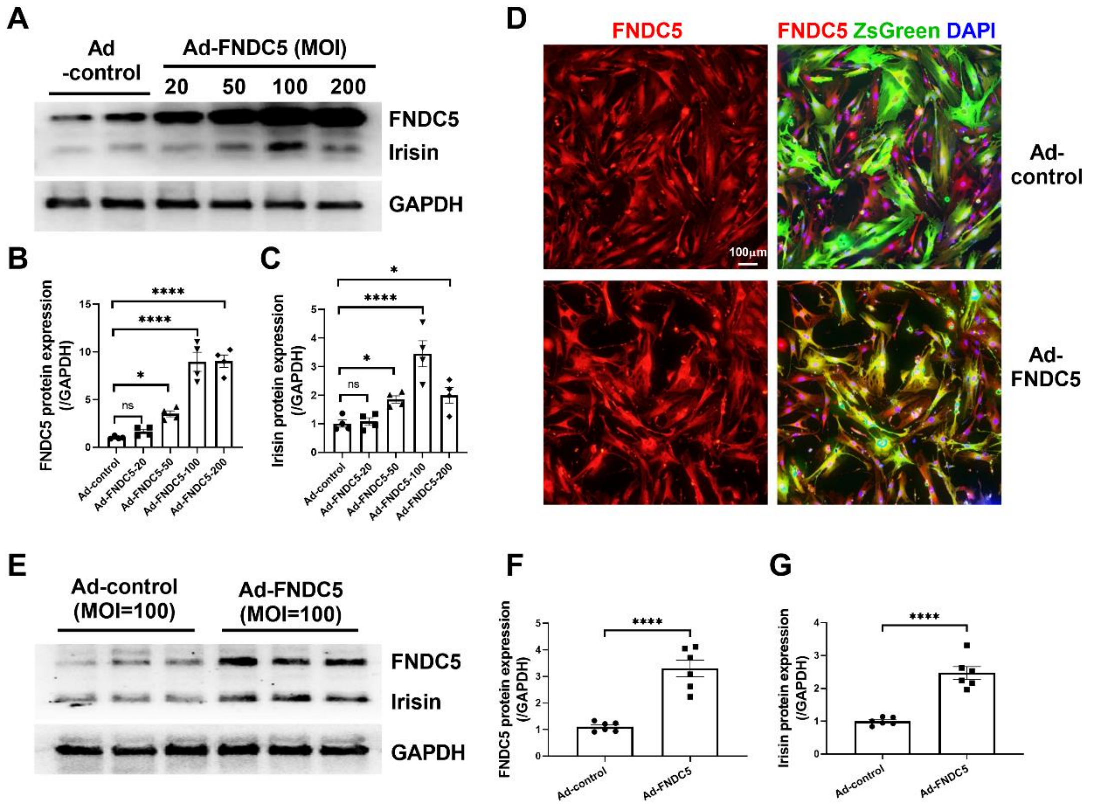


**Figure S4. Adenoviruses harboring FNDC5 increase FNDC5 and irisin protein expression in ADSCs.**

A-C. Western blot (A) and quantification of FNDC5 (B) and irisin (C) in mouse ADSCs 2 days after transfection with control adenovirus (Ad-control) or adenovirus harboring FNDC5 (Ad-FNDC5). Multiplicity of infection (MOI)=20, 50, 100, 200. n=4.

D. FNDC5 immunostaining in mouse ADSCs 2 days after transfection with Ad-control or Ad-FNDC5.

E-G. Western blots (E) and quantification of FNDC5 (F) and irisin (G) in rat ADSCs 2 days after transfection with Ad-control or Ad-FNDC5. MOI=100. n=6.

The data in F and G were analyzed by unpaired, 2-tailed Student's t test. Other data were analyzed by one-way ANOVA, followed by a Bonferroni *post hoc* test. *P<0.05, ****P<0.0001. ns, not significant.

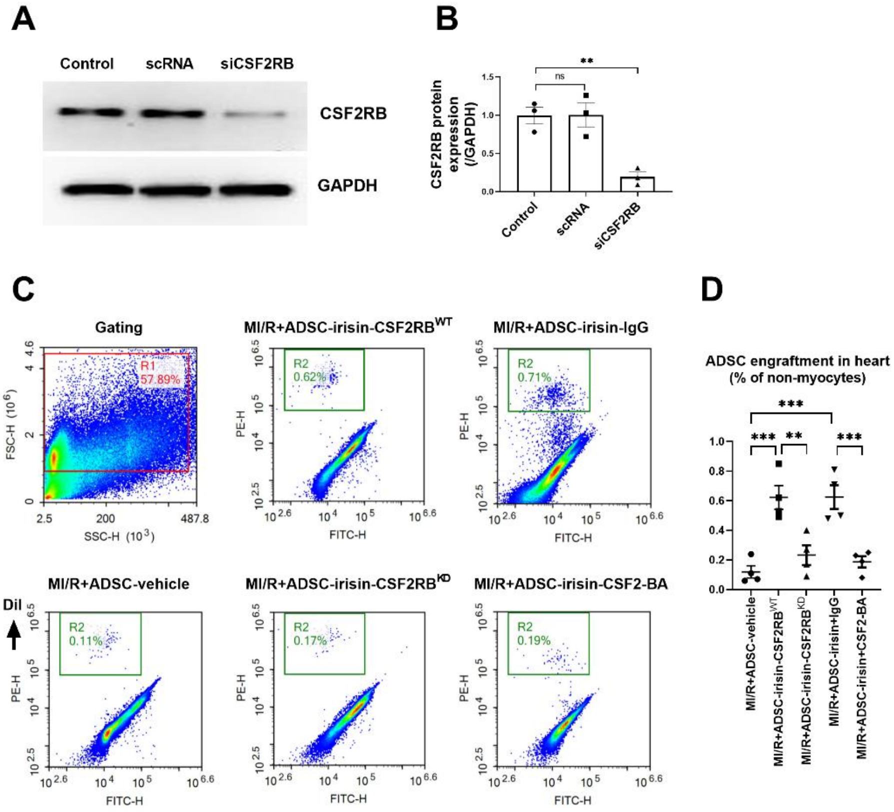


**Figure S5. CSF2/CSF2RB is essential for irisin-increased cardiac homing of intravenously delivered ADSCs.**

A-B. Western blots and quantification of CSF2RB protein expression in ADSCs. ADSCs were transfected with scRNA or siRNA against CSF2RB (siCSF2RB) for 48 hours. n=3.

C. Flow cytometric analysis of CM-DiI-labeled ADSCs in hearts 1 day after intravenous injection (2 days after MI/R).

D. Myocardial ADSC homing was quantified as the ratio of CM-DiI+ cells to the total number of non-myocytes (n=4 mice).

The data were analyzed by one-way ANOVA, followed by a Bonferroni *post hoc* test. CSF2RB$^{WT}$, CSF2RB scRNA. CSF2RB$^{KD}$, CSF2RB siRNA. CSF2-BA, the blocking antibody of CSF2. **P<0.01, ***P<0.001. ns, not significant.

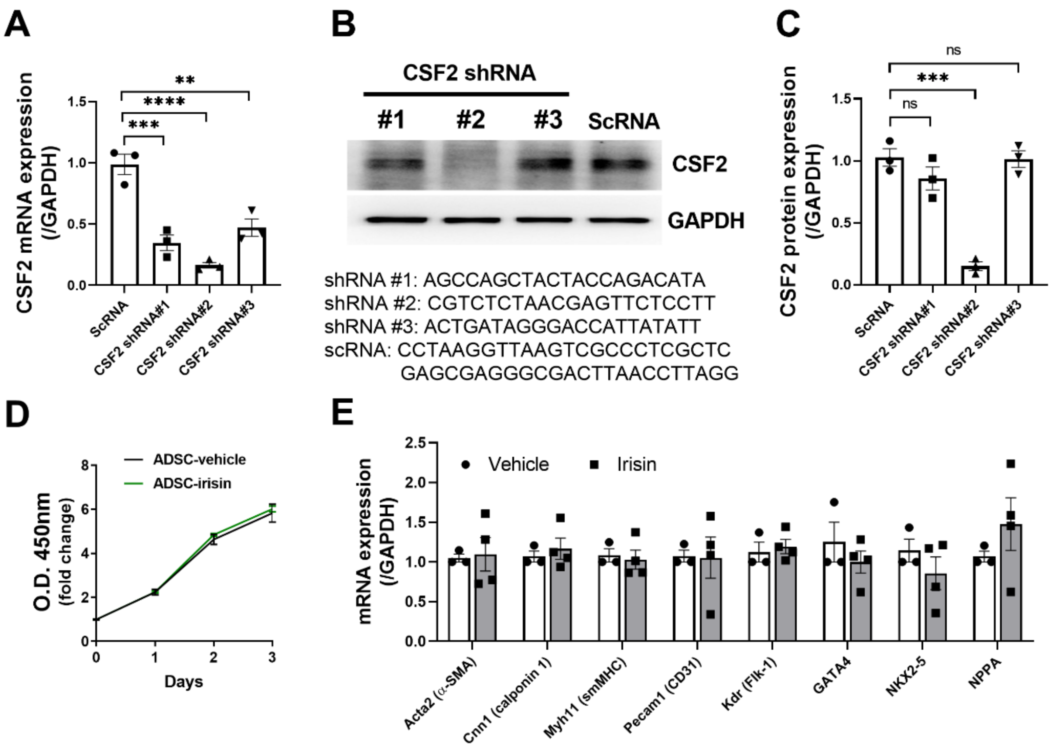


**Figure S6.**

A. Quantitative PCR analysis of CSF2 mRNA expression in mouse cardiac fibroblasts. Primary cardiac fibroblasts were transfected with 3 CSF2 shRNAs for 36 hours. n=3.

B and C. Western blots and quantification of CSF2 protein expression in mouse cardiac fibroblasts. Primary cardiac fibroblasts were transfected with 3 CSF2 shRNAs for 36 hours. n=3.

shRNA, short hairpin RNA. Data were analyzed by one-way ANOVA, followed by a Bonferroni post hoc test. **P<0.01, ***P<0.001, ****P<0.0001. ns, not significant.

D. ADSC proliferation curves (n=8).

E. Quantitative PCR analysis of fibrogenic (α-SMA, calponin 1, smMHC), vasculogenic (CD31, Flk-1), and cardiogenic (GATA4, NKX2-5, NPPA) gene expression in rat ADSCs treated with vehicle or irisin (100 ng/mL) for 7 days. n=3–4.

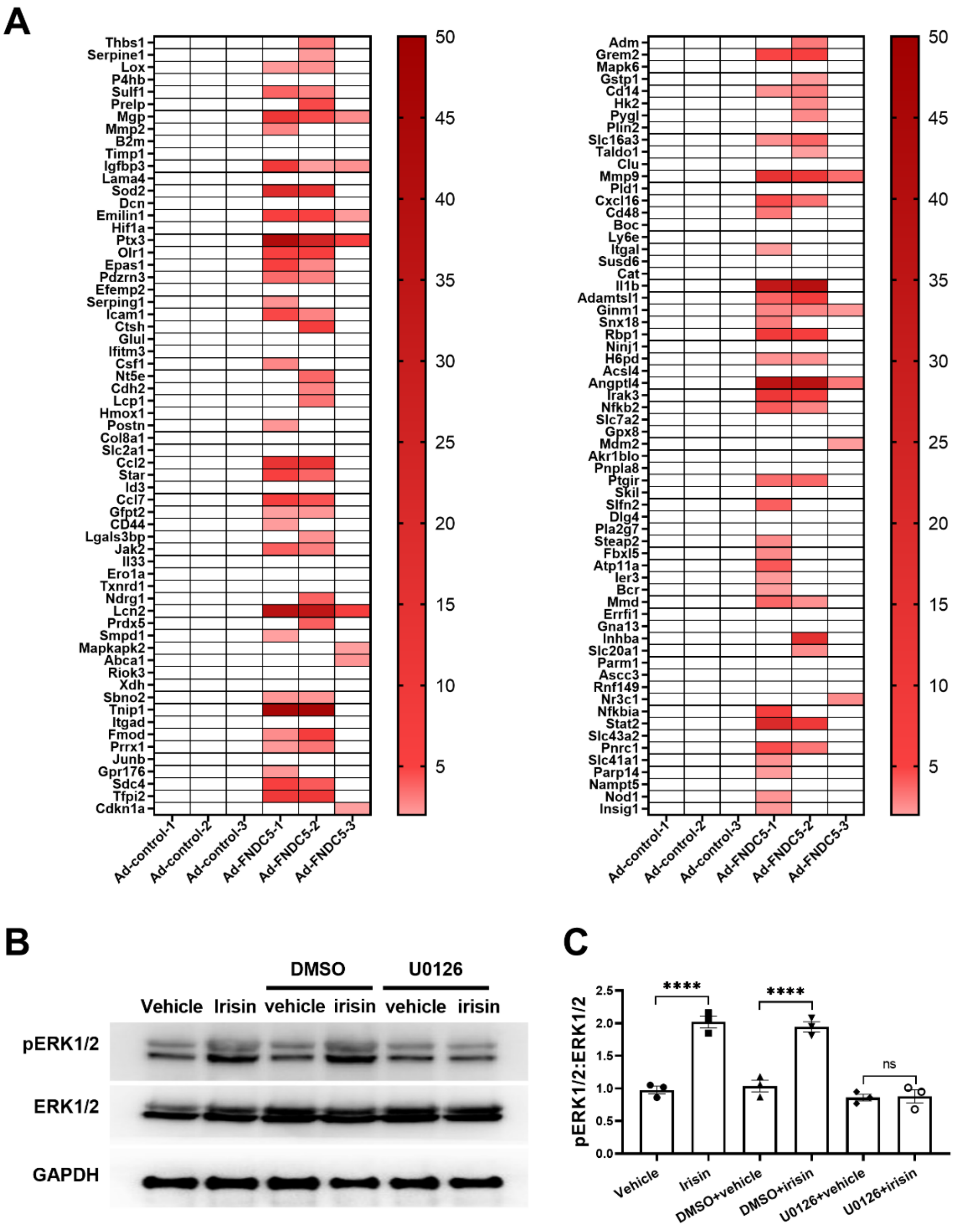


**Figure S7.**

A. Quantitative PCR analysis of mRNA expression for genes of interest (132 genes in total) in rat ADSCs transfected with adenovirus-control (Ad-control) or adenovirus-FNDC5 (Ad-FNDC5). These 132 genes were increased by irisin, as evidenced by RNA sequencing. n=3.

B and C. Western blots (B) and quantification (C) of pERK1/2:ERK1/2 protein expression in ADSCs. ADSCs were treated with recombinant irisin (100 ng/mL) for 30 minutes. ****P<0.0001; ns, not significant.

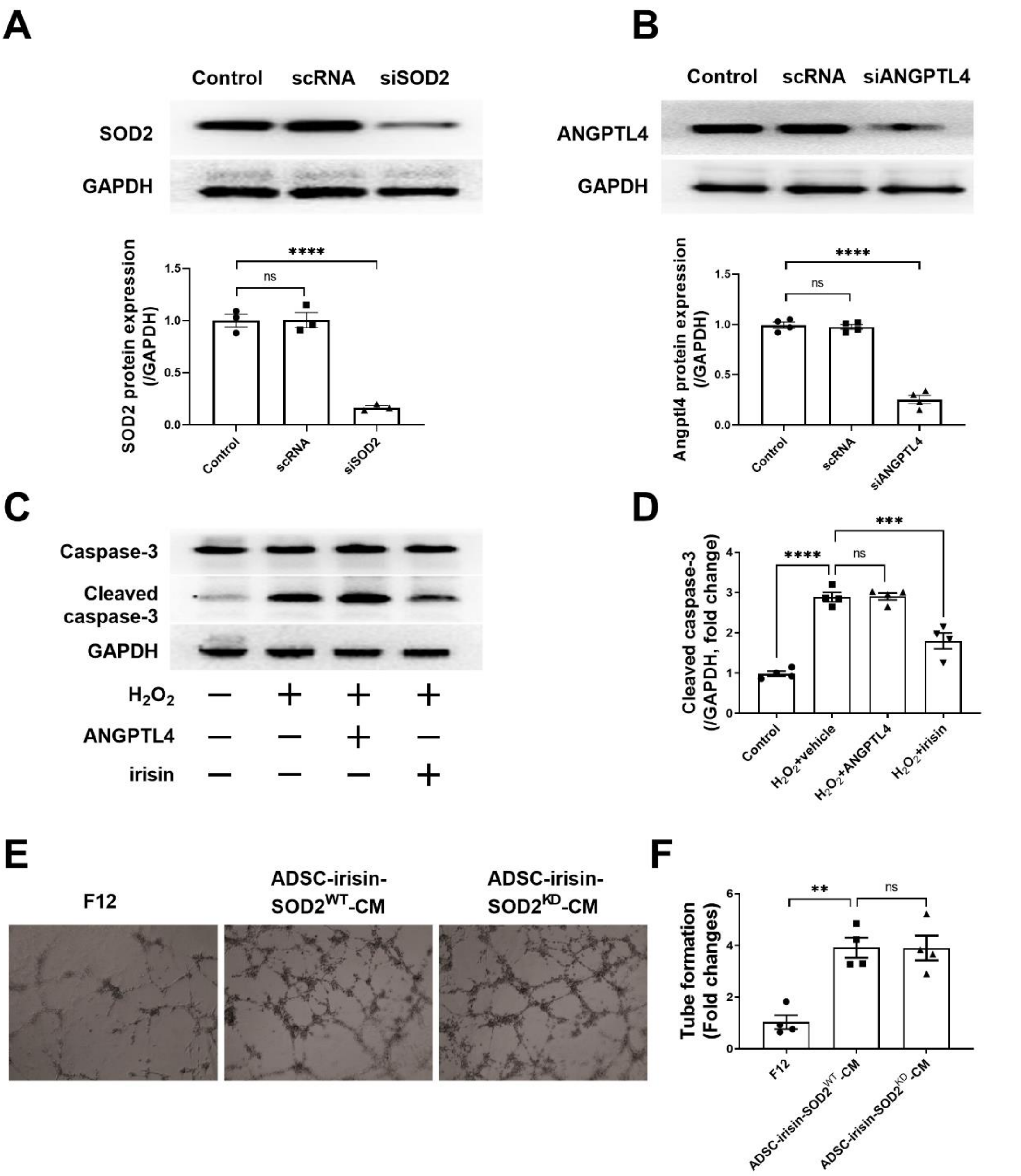


**Figure S8.**

A. Western blots and quantification of SOD2 protein expression in ADSCs. ADSCs were transfected with scRNA or siRNA against SOD2 (siSOD2) for 48 hours. n=3.

B. Western blots and quantification of ANGPTL4 protein expression in ADSCs. ADSCs were transfected with scRNA or siRNA against ANGPTL4 (siANGPTL4) for 48 hours. n=4.

C and D. Western blots and quantification of cleaved caspase-3 protein expression in NRVCs. NRVCs were treated with recombinant ANGPTL4 (2 µg/mL) or irisin (100 ng/mL) 24 hours before $H_2O_2$ (200 µM, 6 hours). n=4.

E and F. Tube formation of rCAECs treated with F12, ADSC-irisin-SOD2$^{WT}$-CM, or ADSC-irisin-SOD2$^{KD}$-CM. n=4. SOD2$^{WT}$, SOD2 scRNA; SOD2$^{KD}$, SOD2 siRNA.

**P<0.01, ***P<0.001, ****P<0.0001; ns, not significant. The data were analyzed by one-way ANOVA, followed by Bonferroni *post hoc* test.

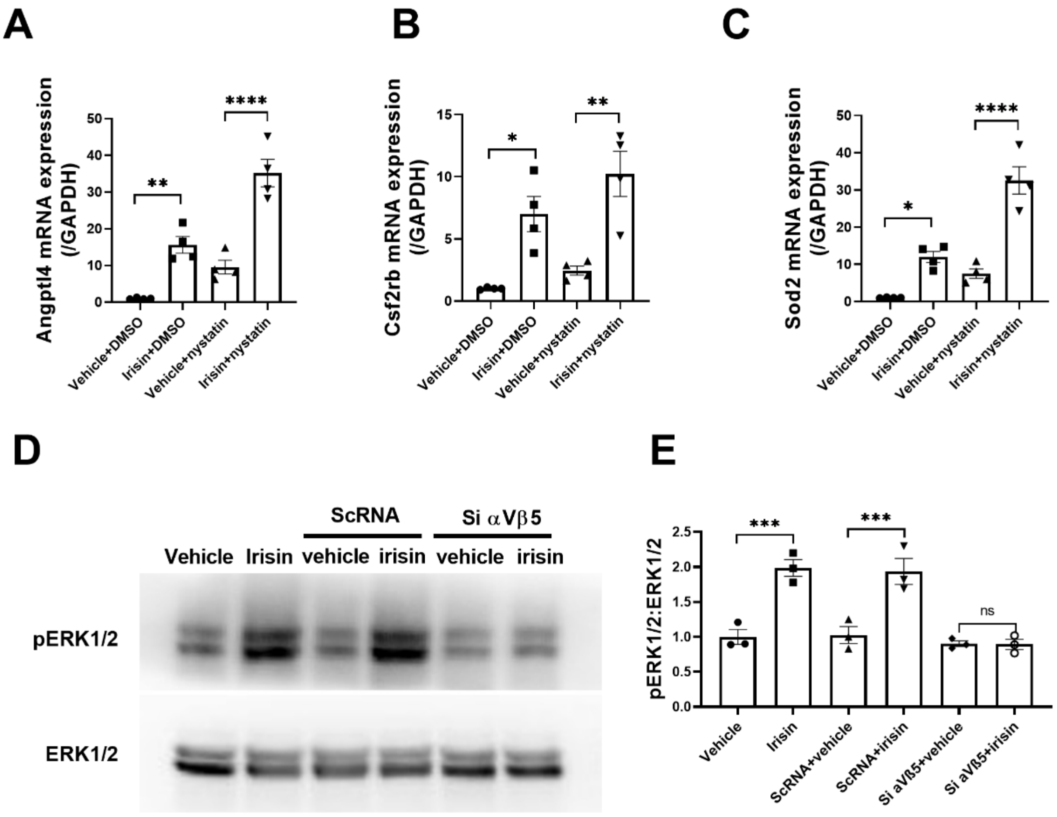


**Figure S9.**

A-C. Quantitative PCR analysis of Angptl4, Csf2rb, and Sod2 mRNA expression in ADSCs. ADSCs were treated with DMSO or nystatin (5 μM) for 12 hours. n=4.

D and E. Western blots (D) and quantification (E) of pERK1/2:ERK1/2 protein expression in ADSCs. ADSCs were transfected with scRNA or siRNAs against integrin αV and β5 (Si αVβ5) for 48 hours and then treated with recombinant irisin (100 ng/mL) for 30 minutes.

*P<0.05, **P<0.01, ***P<0.0001, ****P<0.0001. The data were analyzed by one-way ANOVA, followed by a Bonferroni *post hoc* test.

**Tables**

**Table I:** Genes of the cytokine-cytokine receptor interaction pathway with significantly upregulated expression induced by irisin in rADSCs identified by RNA sequencing (RNAseq) analysis (26 cytokines and 19 cytokine receptors)

| **Cytokines (Gene ID)** | **Fold Change (Irisin/Vehicle)** | **P value** | **Name** |
|---|---|---|---|
| ENSRNOG00000002829 | 79.14075427 | 1.15E-06 | Ppbp |
| ENSRNOG00000022242 | 62.43078777 | 3.00E-32 | Cxcl9 |
| ENSRNOG00000022256 | 12.27842609 | 2.57E-32 | Cxcl10 |
| ENSRNOG00000022298 | 119.9730564 | 6.32E-33 | Cxcl11 |
| ENSRNOG00000024899 | 11.39479722 | 6.88E-27 | Cxcl13 |
| ENSRNOG00000026647 | 6.610628004 | 7.37E-67 | Cxcl16 |
| ENSRNOG00000028015 | 4.060679234 | 1.75E-33 | Pf4 |
| ENSRNOG00000011984 | 3.707080486 | 4.05E-10 | Cxcl14 |
| ENSRNOG00000021851 | 26.81771201 | 0.005086 | Ccl1 |
| ENSRNOG00000015992 | 9.680106888 | 3.32E-07 | Ccl20 |
| ENSRNOG00000007159 | 9.402859941 | 1.42E-14 | Ccl2 |
| ENSRNOG00000011406 | 6.60562448 | 2.75E-08 | Ccl4 |
| ENSRNOG00000011205 | 8.384806719 | 1.43E-10 | Ccl3 |
| ENSRNOG00000000239 | 7.932686519 | 1.07E-42 | Ccl7 |
| ENSRNOG00000010278 | 51.12247342 | 7.51E-119 | Il6 |
| ENSRNOG00000024390 | 2.047011185 | 0.009744 | Osm |
| ENSRNOG00000008525 | 106.5589005 | 1.01E-33 | Csf3 |
| ENSRNOG00000026805 | 7.841771392 | 2.54E-12 | Csf2 |
| ENSRNOG00000058856 | 2.61438999 | 0.015737 | Tslp |
| ENSRNOG00000007027 | 8.676243646 | 4.37E-86 | Hgf |
| ENSRNOG00000004647 | 2.564628005 | 1.08E-05 | Il10 |
| ENSRNOG00000013269 | 4.672494347 | 2.54E-06 | Tnfsf10 |
| ENSRNOG00000002968 | 25.07882016 | 2.69E-06 | Tnfsf4 |
| ENSRNOG00000004575 | 35.29043015 | 6.59E-22 | Il1a |
| ENSRNOG00000004649 | 10.12241892 | 1.60E-08 | Il1b |
| ENSRNOG00000012509 | 85.81245466 | 8.17E-07 | Il17f |
| | | | |
| **Cytokine receptors (Gene ID)** | **Fold Change (Irisin/Vehicle)** | **P value** | **Name** |
| ENSRNOG00000010665 | 10.10604682 | 2.33E-06 | Ccr7 |
| ENSRNOG00000011696 | 2.012087039 | 1.04E-05 | Lifr |
| ENSRNOG00000033192 | 2.070104723 | 4.31E-11 | Osmr |
| ENSRNOG00000001713 | 3.074177331 | 6.03E-06 | Il13ra1 |
| ENSRNOG00000007544 | 47.85899368 | 0.000197 | Il23r |
| ENSRNOG00000000187 | 4.002190092 | 4.50E-22 | Csf2rb |
| ENSRNOG00000047647 | 168.3991568 | 3.27E-36 | Il2ra |
| ENSRNOG00000003954 | 2.191892915 | 7.49E-17 | Il2rg |
| ENSRNOG00000018706 | 8.756001075 | 9.90E-11 | Il15ra |

| | | | |
|---|---|---|---|
| ENSRNOG00000057557 | 18.96913428 | 0.008953 | Prlr |
| ENSRNOG00000003254 | 4.280377418 | 0.006222 | Il23a |
| ENSRNOG00000004332 | 2.183894984 | 2.04E-15 | Egfr |
| ENSRNOG00000016308 | 2.054807912 | 2.00E-07 | Il10ra |
| ENSRNOG00000028638 | 1.214787847 | 0.015096 | Il10rb |
| ENSRNOG00000033984 | 2.645726785 | 7.51E-21 | Ifnlr1 |
| ENSRNOG00000011517 | 4.663927188 | 4.85E-29 | Tnfrsf21 |
| ENSRNOG00000016575 | 6.462754384 | 1.32E-50 | Tnfrsf1b |
| ENSRNOG00000016782 | 3.483062639 | 6.47E-06 | Tnfrsf8 |
| ENSRNOG00000016289 | 3.199449314 | 4.29E-16 | Bmpr1b |

**Table II:** Differentially expressed genes (mean count of >1000) between rADSC-vehicle and rADSC-irisin identified by RNA sequencing (RNAseq) analysis (141 total)

| Gene ID | Fold Change (Irisin/Vehicle) | P value | Name |
|---|---|---|---|
| ENSRNOG00000045829 | 2.123140393 | 2.55E-14 | Thbs1 |
| ENSRNOG00000001414 | 3.794964961 | 1.18E-30 | Serpine1 |
| ENSRNOG00000015036 | 2.553337851 | 3.93E-30 | Ctgf |
| ENSRNOG00000014426 | 2.415426824 | 1.67E-26 | Lox |
| ENSRNOG00000036689 | 2.3827209 | 2.72E-27 | P4hb |
| ENSRNOG00000009037 | 2.422093523 | 1.65E-19 | Sulf1 |
| ENSRNOG00000003120 | 3.088727522 | 4.75E-43 | Prelp |
| ENSRNOG00000005695 | 8.130157994 | 2.02E-13 | Mgp |
| ENSRNOG00000014350 | 2.158863434 | 1.45E-13 | Cyr61 |
| ENSRNOG00000016695 | 3.690135869 | 2.71E-58 | Mmp2 |
| ENSRNOG00000010947 | 2.18906125 | 2.35E-19 | Mmp14 |
| ENSRNOG00000017123 | 4.681066953 | 1.84E-212 | B2m |
| ENSRNOG00000010208 | 6.551981115 | 1.95E-50 | Timp1 |
| ENSRNOG00000061910 | 3.959444431 | 1.04E-05 | Igfbp3 |
| ENSRNOG00000000599 | 2.000325283 | 1.98E-26 | Lama4 |
| ENSRNOG00000019048 | 3.083997642 | 0.000291946 | Sod2 |
| ENSRNOG00000004554 | 9.024900712 | 5.55E-106 | Dcn |
| ENSRNOG00000008246 | 3.096440211 | 1.82E-16 | Emilin1 |
| ENSRNOG00000033192 | 2.070104723 | 4.31E-11 | Osmr |
| ENSRNOG00000008292 | 4.500981915 | 5.22E-63 | Hif1a |
| ENSRNOG00000012280 | 3.856109334 | 7.94E-05 | Ptx3 |
| ENSRNOG00000056219 | 2.520122898 | 3.46E-15 | Olr1 |
| ENSRNOG00000021318 | 2.281308116 | 7.83E-14 | Epas1 |
| ENSRNOG00000057556 | 2.170829645 | 1.74E-14 | Pdzrn3 |
| ENSRNOG00000020587 | 2.096719524 | 3.27E-19 | Efemp2 |
| ENSRNOG00000007457 | 4.272423867 | 4.01E-49 | Serping1 |
| ENSRNOG00000020679 | 2.731897515 | 3.04E-31 | Icam1 |
| ENSRNOG00000014064 | 3.069604668 | 4.61E-47 | Ctsh |
| ENSRNOG00000049560 | 2.129185669 | 4.12E-10 | Glul |
| ENSRNOG00000015078 | 2.51475911 | 9.04E-42 | Ifitm3 |
| ENSRNOG00000018659 | 2.304451584 | 3.87E-32 | Csf1 |
| ENSRNOG00000011071 | 3.982595408 | 6.66E-43 | Nt5e |
| ENSRNOG00000015602 | 2.447127835 | 1.57E-23 | Cdh2 |
| ENSRNOG00000010319 | 2.808347245 | 1.39E-52 | Lcp1 |
| ENSRNOG00000014117 | 5.103462588 | 1.58E-43 | Hmox1 |
| ENSRNOG00000012660 | 2.555956996 | 8.69E-39 | Postn |
| ENSRNOG00000039668 | 2.117682515 | 9.07E-20 | Col8a1 |
| ENSRNOG00000007284 | 2.87683353 | 9.20E-24 | Slc2a1 |
| ENSRNOG00000007159 | 9.402859941 | 1.42E-14 | Ccl2 |
| ENSRNOG00000015052 | 4.078482896 | 2.08E-27 | Star |

| | | | |
|---|---|---|---|
| ENSRNOG00000026124 | 2.205059155 | 2.31E-20 | Id3 |
| ENSRNOG00000000239 | 7.932686519 | 1.07E-42 | Ccl7 |
| ENSRNOG00000002810 | 3.144637479 | 1.76E-15 | Gfpt2 |
| ENSRNOG00000006094 | 2.664137227 | 1.45E-40 | Cd44 |
| ENSRNOG00000003217 | 8.316672588 | 2.07E-258 | Lgals3bp |
| ENSRNOG00000028703 | 2.656715611 | 2.76E-41 | Slc39a6 |
| ENSRNOG00000054978 | 2.011544676 | 1.85E-33 | Hist1h1c |
| ENSRNOG00000059968 | 3.633766332 | 1.93E-64 | Jak2 |
| ENSRNOG00000016456 | 9.306631382 | 2.80E-79 | Il33 |
| ENSRNOG00000019587 | 2.573364088 | 3.10E-27 | Ptprn |
| ENSRNOG00000006462 | 2.406939191 | 3.22E-35 | Ero1a |
| ENSRNOG00000009088 | 2.243466246 | 3.00E-33 | Txnrd1 |
| ENSRNOG00000007393 | 2.470140602 | 8.16E-36 | Ndrg1 |
| ENSRNOG00000013973 | 8.833885756 | 1.74E-09 | Lcn2 |
| ENSRNOG00000021125 | 3.075576105 | 5.25E-18 | Prdx5 |
| ENSRNOG00000017977 | 2.314139777 | 7.96E-16 | Smpd1 |
| ENSRNOG00000004726 | 2.125972599 | 3.15E-16 | Mapkapk2 |
| ENSRNOG00000018126 | 2.056723332 | 1.85E-20 | Abca1 |
| ENSRNOG00000023376 | 3.997382435 | 2.36E-50 | Riok3 |
| ENSRNOG00000007081 | 2.35734155 | 6.07E-20 | Xdh |
| ENSRNOG00000013987 | 2.009129906 | 3.89E-11 | Sbno2 |
| ENSRNOG00000010370 | 3.795503078 | 1.74E-18 | Tnip1 |
| ENSRNOG00000019728 | 2.398785071 | 9.24E-17 | Itgad |
| ENSRNOG00000003183 | 2.813941831 | 6.68E-18 | Fmod |
| ENSRNOG00000003720 | 2.508343112 | 3.24E-29 | Prrx1 |
| ENSRNOG00000042838 | 2.389507841 | 4.57E-13 | Junb |
| ENSRNOG00000005971 | 2.499801359 | 2.50E-23 | Gpr176 |
| ENSRNOG00000014297 | 2.22286935 | 7.75E-25 | Sdc4 |
| ENSRNOG00000010513 | 6.81433998 | 2.27E-58 | Tfpi2 |
| ENSRNOG00000004332 | 2.183894984 | 2.04E-15 | Egfr |
| ENSRNOG00000000521 | 3.301449646 | 1.21E-41 | Cdkn1a |
| ENSRNOG00000027030 | 2.716836302 | 2.23E-29 | Adm |
| ENSRNOG00000003616 | 3.879966629 | 4.31E-43 | Grem2 |
| ENSRNOG00000009381 | 2.589156809 | 5.64E-58 | Mapk6 |
| ENSRNOG00000018237 | 2.021072988 | 4.29E-23 | Gstp1 |
| ENSRNOG00000017819 | 2.988973924 | 1.08E-18 | Cd14 |
| ENSRNOG00000006116 | 2.464474935 | 1.37E-26 | Hk2 |
| ENSRNOG00000006388 | 2.608609931 | 2.00E-20 | Pygl |
| ENSRNOG00000007060 | 2.319236622 | 1.02E-18 | Plin2 |
| ENSRNOG00000036677 | 2.712847435 | 3.51E-20 | Slc16a3 |
| ENSRNOG00000018367 | 2.212776124 | 4.41E-15 | Taldo1 |
| ENSRNOG00000016460 | 4.569960126 | 1.55E-47 | Clu |
| ENSRNOG00000017539 | 2.543673393 | 0.011815667 | Mmp9 |
| ENSRNOG00000028156 | 2.248601129 | 2.95E-28 | Pld1 |
| ENSRNOG00000026647 | 6.610628004 | 7.37E-67 | Cxcl16 |
| ENSRNOG00000004737 | 2.078370915 | 4.06E-25 | Cd48 |

| | | | |
|---|---|---|---|
| ENSRNOG00000002041 | 2.994542165 | 1.08E-14 | Boc |
| ENSRNOG00000007091 | 7.239120152 | 1.23E-164 | Ly6e |
| ENSRNOG00000017980 | 3.965036211 | 0.00027917 | Itgal |
| ENSRNOG00000045941 | 2.267418248 | 4.64E-17 | Susd6 |
| ENSRNOG00000008364 | 2.167035481 | 5.60E-43 | Cat |
| ENSRNOG00000004649 | 10.12241892 | 1.60E-08 | Il1b |
| ENSRNOG00000006956 | 2.75533061 | 2.34E-25 | Adamtsl1 |
| ENSRNOG00000015239 | 2.537974234 | 2.87E-43 | Ginm1 |
| ENSRNOG00000010971 | 3.792222643 | 1.16E-23 | Snx18 |
| ENSRNOG00000013794 | 3.009467626 | 8.83E-15 | Rbp1 |
| ENSRNOG00000016587 | 3.212489649 | 2.58E-31 | Ninj1 |
| ENSRNOG00000017523 | 2.942902216 | 1.65E-14 | H6pd |
| ENSRNOG00000019180 | 3.026206776 | 1.08E-18 | Acsl4 |
| ENSRNOG00000007545 | 10.61149297 | 8.86E-07 | Angptl4 |
| ENSRNOG00000004226 | 2.450488003 | 5.57E-07 | Irak3 |
| ENSRNOG00000021243 | 2.503333796 | 3.17E-15 | Siglec1 |
| ENSRNOG00000019311 | 2.414601263 | 6.96E-14 | Nfkb2 |
| ENSRNOG00000011016 | 3.843280555 | 3.21E-48 | Slc7a2 |
| ENSRNOG00000010461 | 2.118692675 | 6.94E-20 | Gpx8 |
| ENSRNOG00000006304 | 2.505740465 | 1.75E-43 | Mdm2 |
| ENSRNOG00000009734 | 9.618816039 | 1.94E-153 | Akr1b10 |
| ENSRNOG00000009263 | 3.259227328 | 3.45E-61 | Ifi27 |
| ENSRNOG00000039091 | 2.009605377 | 3.59E-06 | Pnpla8 |
| ENSRNOG00000016756 | 2.141057573 | 2.85E-31 | Ptgir |
| ENSRNOG00000009899 | 2.056766989 | 1.58E-30 | Skil |
| ENSRNOG00000037113 | 3.203659436 | 3.91E-37 | Slfn2 |
| ENSRNOG00000018526 | 2.286855202 | 3.10E-23 | Dlg4 |
| ENSRNOG00000025691 | 2.143018598 | 1.52E-22 | Pla2g7 |
| ENSRNOG00000006076 | 2.078669801 | 7.48E-11 | Steap2 |
| ENSRNOG00000005261 | 3.229041205 | 7.99E-49 | Fbxl5 |
| ENSRNOG00000017154 | 3.123241958 | 5.28E-57 | Atp11a |
| ENSRNOG00000000827 | 2.830483379 | 3.92E-20 | Ier3 |
| ENSRNOG00000001304 | 2.461858462 | 1.84E-09 | Bcr |
| ENSRNOG00000002436 | 3.933758869 | 1.06E-52 | Mmd |
| ENSRNOG00000058186 | 2.129961253 | 2.32E-05 | Errfi1 |
| ENSRNOG00000036745 | 2.107358003 | 1.21E-11 | Gna13 |
| ENSRNOG00000018911 | 4.551977261 | 4.85E-32 | Pfkfb3 |
| ENSRNOG00000014320 | 7.982588122 | 1.11E-12 | Inhba |
| ENSRNOG00000018567 | 4.232721985 | 7.64E-35 | Slc20a1 |
| ENSRNOG00000002579 | 4.41787323 | 7.62E-82 | Parm1 |
| ENSRNOG00000037604 | 2.267769699 | 2.22E-23 | Ascc3 |
| ENSRNOG00000003486 | 2.433506321 | 2.01E-11 | Mnda |
| ENSRNOG00000013946 | 2.004282042 | 5.84E-08 | Rnf149 |
| ENSRNOG00000014096 | 2.175787918 | 2.27E-14 | Nr3c1 |
| ENSRNOG00000007390 | 5.492658517 | 2.19E-08 | Nfkbia |
| ENSRNOG00000031081 | 3.578701679 | 3.69E-42 | Stat2 |

| | | | |
|---|---|---|---|
| ENSRNOG00000003835 | 2.003788172 | 6.40E-21 | Slc43a2 |
| ENSRNOG00000007793 | 2.36638579 | 8.08E-14 | Pnrc1 |
| ENSRNOG00000004581 | 2.016797483 | 2.72E-21 | Zdhhc9 |
| ENSRNOG00000042320 | 2.251495072 | 5.00E-20 | Slc41a1 |
| ENSRNOG00000023334 | 2.99500111 | 2.09E-25 | Parp14 |
| ENSRNOG00000009754 | 2.320730631 | 1.10E-40 | Nampt |
| ENSRNOG00000010629 | 2.684884951 | 9.33E-26 | Nod1 |
| ENSRNOG00000006646 | 2.632162641 | 1.22E-37 | Vopp1 |
| ENSRNOG00000006859 | 2.130044087 | 6.51E-07 | Insig1 |

**Table III:** ADSCs expressed 17 α subunits and 8 β subunits of the integrin family

| Rat ADSCs | | | Mouse ADSCs | |
|---|---|---|---|---|
| Name | | baseMeanA | Name | baseMeanA |
| Integrin α | Itga11 | 22099.33333 | Itga5 | 12322.87277 |
| | Itga8 | 8705.333333 | Itgav | 5350.083136 |
| | Itga5 | 8095.333333 | Itga11 | 2434.786535 |
| | Itga1 | 4781.666667 | Itga9 | 1612.118898 |
| | Itgav | 4534.333333 | Itgam | 1197.078009 |
| | Itgad | 2485 | Itgax | 502.7084217 |
| | Itgal | 1760.333333 | Itga2 | 387.9117392 |
| | Itgax | 1278.666667 | Itga1 | 374.3388785 |
| | Itga3 | 1113 | Itga4 | 345.0812506 |
| | Itga2 | 479.6666667 | Itgal | 308.1341378 |
| | Itga6 | 458.3333333 | Itga3 | 292.7618817 |
| | Itga9 | 194.3333333 | Itga6 | 272.4367632 |
| | Itga4 | 145.3333333 | Itga7 | 219.8717863 |
| | Itga7 | 49 | Itga10 | 46.71271689 |
| | Itgae | 45 | Itga2b | 44.70433438 |
| | Itga2b | 24.66666667 | Itga8 | 13.88560399 |
| | Itga10 | 1.666666667 | Itgae | 8.976568867 |
| Integrin β | Itgb1 | 58930.33333 | Itgb1 | 24950.84227 |
| | Itgb2 | 5239.666667 | Itgb5 | 6777.456912 |
| | Itgb5 | 1597.666667 | Itgb2 | 5249.732476 |
| | Itgb3 | 323 | Itgb3 | 548.8640977 |
| | Itgb8 | 96 | Itgb7 | 107.5939095 |
| | Itgb4 | 49 | Itgb8 | 71.40077486 |
| | Itgb6 | 4.333333333 | Itgb4 | 45.77376534 |
| | Itgb7 | 1.666666667 | Itgb6 | 0.995259075 |

**Table IV:** Real-time PCR primers

| Genes | Species | Forward primer (5'—3') | Reverse primer (5'—3') |
|---|---|---|---|
| Ccr7 | Rat | GCTGGTGGTGCTGACATACA | AGCAACATCCCGCTGAAGAA |
| Lifr | Rat | TTCTGCCGGTTCATCTCCAC | ACCATGACGAGTTGCACCAT |
| Osmr | Rat | GGCTATGGTAGATGACGCCC | AGTTGCTCTCCACGGATTGG |
| Il13ra1 | Rat | CAAGCCCGGACACCAACTAT | GGAGGACCGGGTTTCACATT |
| Il23r | Rat | GCTTTTCGGAACCTCATGCC | GGTGCAAGTCATGTTGCCT |
| Csf2rb | Rat | GGTTGGGGACTACTGCTTCC | CAGGCTGGCTATTGTCCCAA |
| Il2ra | Rat | ACACAATGGCACATCGGGAA | AAAGTGACCACACCATCGCA |
| Il2rg | Rat | TACTGGCCTCCCCCATGTTA | GGCATTCTCAAGTAGGGCGT |
| Il15ra | Rat | GTGACCTGGGTGTAGACTGC | CCTTTGGGTTCACTTTGCCG |
| Prlr | Rat | ATTATGGTCTGGGCAGTGGC | TTGCGATGGTGGTAGAACCC |
| Il23a | Rat | GCTTTTCGGAACCTCATGCC | GGTGCAAGTCATGTTGCCT |
| Egfr | Rat | CTCTGACGGGCTTTGTCACT | CCTCACCATGAGGCAACCTT |
| Il10ra | Rat | CAGGGCATCCTAAGCACACA | AGGCTTCTGCCAAGGGAATC |
| Il10rb | Rat | ATGGGCTGCATCTGGTTGTT | TCCTTGCATCTCCACACACG |
| Ifnlr1 | Rat | GTCTAGCCCCACCCAGAAAC | TCAGGCACATCATGGGACAC |
| Tnfrsf21 | Rat | CAGTGAGGCGGGTGTAGTTT | GCTGCCACAAGGAGAGGAAT |
| Tnfrsf1b | Rat | ACAGAACCGAGTGTGTGCTT | GCCAGGATGCTACAAATGCG |
| Tnfrsf8 | Rat | CTGACCCTGAACCTGTGACC | ACCAGACCCCTGACAGTCTT |
| Bmpr1b | Rat | CAGAGTGCTGGGCGCATAAT | TGTCTGTCCAGCTTGCTCTC |
| Thbs1 | Rat | AGGCCAAAGACCGGTTTGAT | TGCCAGGCTGGTTATGATCG |
| Serpine1 | Rat | CAACCCACTACGCCTTCACT | TCTGTCTATCTGCTGCCCCT |
| Ctgf | Rat | ACCCAACTATGATGCGAGCC | GATGCACTTTTTGCCCTTCTTA |
| Lox | Rat | CAGGCACCGACCTGGATATG | ACTGGCCAGGCAGTTTTCTT |
| P4hb | Rat | GGTGGACTCAAGCGAAGTGA | AAGAGGACCACCCCATCCTT |
| Sulf1 | Rat | TCCAAGTGAGAGTTGCAGGC | CTGTCCTTTGACGCTTCCCT |
| Prelp | Rat | CCTGGACAACAACCGCATTC | AGCTTGCTGAATACTCCGGG |
| Mgp | Rat | CCAGGAAAGAGTCCGGGAAC | TCTTATTTGGCTCCTCGGCG |
| Cyr61 | Rat | GCACCTCGAGAGAAGGACA | CTGGTCAAGTGGAGAAGGGTG |
| Mmp2 | Rat | AGAGGATACCCCAAGCCACT | CAGGAGTCTGCGATGAGCTT |
| Mmp14 | Rat | TCCATAGGTGGGTCCCTTGA | AGCAGATGACCCCATTTGGC |
| B2m | Rat | GTGTACTCTCGCCATCCACC | GCTCCTTCAGAGTGACGTGT |
| Timp1 | Rat | TGCTCAAAGGATTCGACGCT | AGCAGGGCTCAGATTATGCC |
| Igfbp3 | Rat | GGCCTGACCTACTTGGGAAC | CGCACCGTTATTTGCGACAT |
| Lama4 | Rat | CTGTGTTTTACGTTGGCGGG | CAGCCCTGCTCTGAGTGAAA |
| Sod2 | Rat | CAGACCTGCCTTACGACTATGG | CTCGGTGGCGTTGAGATTGTT |
| Dcn | Rat | AAACTCCTCAGAGTGCCTGC | CAATACCGGACAGGGTTGCT |
| Emilin1 | Rat | CTCAATCTTACCGCAGCCCA | GCCCCCAAACACACTGAAAC |
| Hif1a | Rat | TGGTGGCTCAGCAGTCTATTT | GTGCTGTGATCTGGCATTCG |
| Ptx3 | Rat | ACCTCGCTTCTTTCCCTGTG | CATGGTGTGGGGTCCTCG |
| Olr1 | Rat | CATGGGCCCTTTAACTGGGA | GAAACGCCCCTGGTCCTAAA |
| Epas1 | Rat | GGGGTTAAGGAACCCAGGTG | TGTGTTCGCAGGAAGCTGAT |
| Pdzrn3 | Rat | GGCCCTGTGTGGACAATCAA | ATCTTGGTTCGCGGTGTTCT |
| Efemp2 | Rat | TTTCAGATCCGTGCTGGGAA | CCAACATGACCCTTCCCTGAG |
| Serping1 | Rat | CGCCTCTCTGAGCCTGTATG | GGCACTCAAGTAGACGGCAT |
| Icam1 | Rat | CGGTGCTCAGGTATCCATCC | CTGTCTTCCCCAATGTCGCT |
| Ctsh | Rat | CACCAGTGAAGAACCAGGGG | TCCTTGGCAGCCATGATTGT |
| Glul | Rat | CCTTTCGGCTGGCCTTCTAA | TCGCAGGGTCCTATCTGGAA |
| Ifitm3 | Rat | CCGTGATCAACATGCCCAGA | GCCTGGGCTCCAGTCATATC |
| Csf1 | Rat | AATCACGAGGACCCACAACC | TCTGTCAGTCTCTGCCTGGA |
| Nt5e | Rat | TGCATCGATATGGCCAGTCC | TTTGGAATCCATCCCCACCG |
| Cdh2 | Rat | ATAGCCCGGTTTCACCTGAG | CCCAGGCTTTGATCCCTCTG |
| Lcp1 | Rat | TCATCGATGCCATTCAGCCA | GCTTGCTCCTGTGGGACTTA |

| | | | |
|---|---|---|---|
| Hmox1 | Rat | ACCAGAGTCCCTCACAGACA | TAAATTCCCACTGCCACGGT |
| Postn | Rat | GGTGTGAGGGAGACAGCATC | GGTCCGTGAAAGTGGTTTGC |
| Col8a1 | Rat | TAAACCCTGTGAAGAGACGGG | TGGCAGAGGCTTGATTCCAT |
| Slc2a1 | Rat | GGCCTCTACTGCTCAGTGTC | CGGAACAGCTCCAAGATGGT |
| Ccl2 | Rat | TCCTCCACCACTATGCAGGT | TCCAGCCGACTCATTGGGAT |
| Star | Rat | TGGCTGCCAAAGACCATCAT | GAACTTCCAATGGCGTGCAG |
| Id3 | Rat | ATCTTGCCACAGCCCTCTTC | CTCGACACCCCATTTTCGGA |
| Ccl7 | Rat | TGGACTACGGTCCTAAGGGG | GGAACAAAGTCAATGGGGCG |
| Gfpt2 | Rat | CTGACACCGAGTCCCCTCTA | CTATGAGTAGGGAGCCGGGA |
| Cd44 | Rat | ACAACTTCTGGTCCTGCGAG | TCCCCGTTGAGTTCACTTGG |
| Lgals3bp | Rat | CTCTGTGCCCACACACTGAT | GGTGACAAAAAGCCGTCCAC |
| Slc39a6 | Rat | GTCAGCTCCGTTGGGTAACA | CCAGCAGGGAACCAATGCTA |
| Hist1h1c | Rat | GAAGCCAAGCCCAAAGCAAA | CTCTTGGGGGCCTTCTTAGC |
| Jak2 | Rat | GGGTGCCCAGACGAGATTTA | GCCATGTGGCTACAGAGCTT |
| Il33 | Rat | CCCGCCTTGCAAAATCACAA | CAACTGATGCAGCAAACGCT |
| Ptprn | Rat | CCTGCTGTTGTTGAGCGGC | CCTTGTAAGCGCTGGAGAACT |
| Ero1a | Rat | CGGAAAGCGGACCCAGTTAT | AACAAGCACATTCCAACCGC |
| Txnrd1 | Rat | TTGGCAAATTTCCGGCAGTG | CGCACTTAGACCAGCAGACA |
| Ndrg1 | Rat | CAACATTTTGCGGTCTGCCA | CCCCATGCCAATGACACTCT |
| Lcn2 | Rat | CCGATGAACTGAAGGAGCGA | CAGACAGGTGGGACCTGAAC |
| Prdx5 | Rat | CTCTTTGGGAATCGTCGGCT | GGCCCTCAGGTAAGGAGGTA |
| Smpd1 | Rat | GCGATCCAGTCCTGTCTACG | GAACCCAGAGAACCGTGGAG |
| Mapkapk 2 | Rat | GGCTTTATTCTGCCCTTGGC | AACAACCTCCACAATGCCCT |
| Abca1 | Rat | CGTTGCTTGACAGCTTAGGG | CACAGACGATTCGGGACACA |
| Riok3 | Rat | TGAAGGAAGTCCACCCATGC | AATTGCACACATCTGGCACG |
| Xdh | Rat | AGCTCAGCACGGAGATAACG | GGAATCCTGGTGCGGTACAA |
| Sbno2 | Rat | ATCGGTCTGGAGTCTCGGAA | GGCCCTCATTCTCGTCTAGC |
| Tnip1 | Rat | CACACTCACTGGGCACATCT | TTCCATGAGGGTGGCTCAAC |
| Itgad | Rat | GGTGGGAGGTCTGCTGTTAC | AGGCAGGAGAGTGGATTCCT |
| Fmod | Rat | CTTGGGATCGGAGATCGGG | GTGGACTTCTGTCACGTGCT |
| Prrx1 | Rat | ACCGAAGCTGGGAGAAAGTT | TGGTCTTCCCTCCAATCCCA |
| Junb | Rat | AGAGCCTCCCTTGCTCCATA | TGGAAGGGGGCCATGTAAAC |
| Gpr176 | Rat | ATCTCTGACGCCAAGTCTCG | TGACCGTGGTGACGTTGTAG |
| Sdc4 | Rat | CGAGATCGAGCTGGAGTGAC | AATTGTGTGGCCAGTGCAAC |
| Tfpi2 | Rat | TCCACAGTCGGAAACTCTGC | TTCCTCGGGGAACACATTCG |
| Cdkn1a | Rat | GATCCTGGTGATGTCCGACC | GCGAAGTCAAAGTTCCACCG |
| Adm | Rat | ACTAGGCAGAACAACTCCAGC | GCTCCGATACCCTGCTGAAA |
| Grem2 | Rat | TGTCCCTGTCAGCAGCAAAT | ATCTTCTGTGTCCGATCCGC |
| Mapk6 | Rat | CAGGAGCTTCTCAGCGTGAT | ATGGGAAAGTGCTTCCTCGG |
| Gstp1 | Rat | GCTATGCCACCGTACACCAT | AAAGCCCTAAAGAGCGACCC |
| Cd14 | Rat | GTTGGGCGAGAAAGGACTGA | GCTCCAGCCCAGTGAAAGAT |
| Hk2 | Rat | GAGAGATCGACATGGGCTCG | TCTCGAAGGAGCCAGTGGTA |
| Pygl | Rat | GCAGACTACGAAGCCTATGTCA | TTGACCCCATTGCTGGACTC |
| Plin2 | Rat | GGTCAGTGTGTGAGATGGCA | ATCCTTTGCCCCAGTTACGG |
| Slc16a3 | Rat | CTCTTTGTGCCTCCGGTCTT | AGGTAGACAGAGTAGGGCCG |
| Taldo1 | Rat | CCACTGCAACATGACACTGC | CACCCGTGTTACGGAAGGAA |
| Clu | Rat | AAGCCGCATGGAATGAGACA | AGAGCATCAAGTGCAGGCAT |
| Mmp9 | Rat | TCCAGCATCTGTATGGTCGTG | GCAGTGGGACACATAGTGGG |
| Pld1 | Rat | AGAAAGCTGCCTTGAGGGTC | AGTGCCTAGTGTGGGTAGGT |
| Cxcl16 | Rat | CTTCTGGCACCCAGATACCG | ACTCTGCCCATGACCGATTC |
| Cd48 | Rat | AAGCACCCACTTGCATCGTA | ACTGGTCCTCAGTTTCGTGC |
| Boc | Rat | CAGCAGACTACACGGAAGCA | AGCAGCTGGTGAGTTGAGTC |
| Ly6e | Rat | TGTCAACCTTGGCTACACCC | CAAGCTGAGCAGGAGTCCAA |
| Itgal | Rat | CCCAGGCTGCAGTTATCACA | AACGCATGCCCTAACCTCAA |

| | | | |
|---|---|---|---|
| Susd6 | Rat | CTCTGGAGTCGGTCACAGC | ATGACACCGATCTTCTGGGA |
| Cat | Rat | CTGACTGACGCGATTGCCTA | GTGGTCAGGACATCGGGTTT |
| Il1b | Rat | CCTTGTCGAGAATGGGCAGT | TTCTGTCGACAATGCTGCCT |
| Adamtsl1 | Rat | GATACCTTCAGAGCAGCCCTC | CACGTGGATTTCAGCGAAGA |
| Ginm1 | Rat | GGTTGTGGAGCTCGATGGAA | TTGGAAAAATCGGGAGGCGT |
| Snx18 | Rat | TGCCTTCAGACCGGTTGTTT | CCAGCATCTTACCAGGCGAT |
| Rbp1 | Rat | TGACAGGCATAGATGACCGC | TTGGGCCATCCCACTTGTTC |
| Ninj1 | Rat | GACCCCTGCAACTGCTGTAT | TGGGTTTCGGTGGAACTGTC |
| H6pd | Rat | GCAGACATTCACTGCCCTCT | AGACAGAGCCATCCTGCCTA |
| Acsl4 | Rat | AGTTCGATTGAGCCCAGAGC | AACAGAGAGATCAACAAAGGCT |
| Angptl4 | Rat | ACCCGAAGGATAGAGTCCCC | GCCATCTTGGGAAGCCTCTT |
| Irak3 | Rat | AGAATTGCTGTGGTCCTGGG | GTCCGGGGTCACTTTCTCTG |
| Siglec1 | Rat | TGGAGATCCAGAAGCCGGTA | CCAAGAAAAGTTGGCAGGGC |
| Nfkb2 | Rat | ACGCTCTGCCCCAAATGTTA | CTAGATGCAGTGGGGTTCGG |
| Slc7a2 | Rat | TGGGTTGTCATGGGTTTGCT | TCCCAAGCACACATGTACCC |
| Gpx8 | Rat | ATGGTTCTGTGCACCGTGAT | TCTTGTCTGTGAACCGGCAG |
| Mdm2 | Rat | GCCTGAGTGATGAAGGGCAT | CCAGTTCTCACGAAGGGTCC |
| Akr1b10 | Rat | CATCGTGGTCACAGCCTACA | CTCTTGTATGCGGGAGGGTG |
| Ifi27 | Rat | GCTCCTATCTGGAACGCACA | CAGTGCAGTCTGGGCTAACA |
| Pnpla8 | Rat | CCCCTAGATGAAAGCCGCAA | TGATGGCCTTGAGGAACACA |
| Ptgir | Rat | GGGAACTGGGAGTTGACTGG | GCAGGTCCTACCGAGTCTTG |
| Skil | Rat | GTCCGCCAGAACACTGGTTA | ATGGTGGGCAGACCTTTCAG |
| Slfn2 | Rat | TGTCACCAGGAGGATTTGGC | CCGTGTTTGCGAATGCAGAA |
| Dlg4 | Rat | AGGAGTACCCGCTGTAGGG | TCAGGAGAGCCCCCATGC |
| Pla2g7 | Rat | AGCTCGGAAAGAGCAGGTTC | GGATCAAGGGCAATCCCACA |
| Steap2 | Rat | TGTTCTTGCGCTTGTCTTGC | GACCCTCTCAGGGGACAGAT |
| Fbxl5 | Rat | AGCATACAGCTCTGCAGTCTC | CCTGCACTTTTCAGAACGCC |
| Atp11a | Rat | TCTTCACTGACAAGACCGGC | ACATCGTCCCCACAATGGTC |
| Ier3 | Rat | ATGCAGGGGTGCGAGATATG | GCCACAGACCGTATTCCCAA |
| Bcr | Rat | ATGACCCGGAAACCTGTGAC | GGAAAGGTACAGTCTGGGGC |
| Mmd | Rat | CAAGCGCCGTTTCTATCACG | GAATGAGGAACCGTGGTGGT |
| Errfi1 | Rat | CTCCCGTACCCCAAGTCCTA | ATGGCGGTCTCTCAGGTAGT |
| Gna13 | Rat | GGAATGCTTCCGGGGGAAA | ACAGCGAGTCTCCTCATCAC |
| Pfkfb3 | Rat | CGCAATAGTGTCACCCCACT | GGGGGCTCCTCATGTTCTTT |
| Inhba | Rat | GACCTCATGAGACAAGAGCC | AAGTCGTGTGGTTGCCTTCT |
| Slc20a1 | Rat | TCCTCTGTGGGGTACCATCC | TTCTCAACATCGCCAGGAGC |
| Parm1 | Rat | CACCAGTCCGCCTTTGAAGA | GAGGTAACGGAAGCGGAGAG |
| Ascc3 | Rat | TGGCTGTAAAACGCGTAGGA | TGGCCTGTTTGGGTCACAAT |
| Mnda | Rat | CCCCGAGTATAAAGGCGGTC | TCACTGGCCAGCAATGACTT |
| Rnf149 | Rat | CACAGGACCTACCCATCCCA | AAGACGGCACTAGCAGATGG |
| Nr3c1 | Rat | TGTGAAAATGGGTCGGTGCT | ACGGTTAATCTGCACAGCCT |
| Nfkbia | Rat | AGCCCCGAGCATTCTATTGT | CACACTGGGGTCAGCTACAG |
| Stat2 | Rat | CCTTGTGAGCAGGGTTAGCA | CTGCCCTTAAGACCCCTGTG |
| Slc43a2 | Rat | CACCACACTGGAGCCCATAC | ATGCTTGCTGGAGAAGTCCC |
| Pnrc1 | Rat | TATCGCAGCATCCGACACAA | CGTGCCTTAGCACCCACTTA |
| Zdhhc9 | Rat | CATCGTCTATGTGGCCCTCAA | TGTACGGATTCTGCACACGG |
| Slc41a1 | Rat | GGCAGGAGAAAGTCGCTTCA | CACTCGGACCTCTGGAAACC |
| Parp14 | Rat | ATCAGCCACCGCTCAAATCA | TCGGGGAAACACTCAACTCG |
| Nampt | Rat | TGGGGTGAAGACCTGAGACA | TGGCAGCAACTTGTAGCCTT |
| Nod1 | Rat | TCAGCAATGAAAGGCGGGAT | TCCGAATGTTGGTGACCAGG |
| Vopp1 | Rat | TTCCAGGTCCAGCCCAATTC | CAAAAGGGCCTGTCCATCCT |
| Insig1 | Rat | CACGTCTGGAGCTACCCAAG | CGCCAAATGAGAAGAGCACG |
| Csf2rb | Mouse | GGTTGGGGACTACTGCTTCC | CAGGCTGGCTATTGTCCCAA |
| Angptl4 | Mouse | CACCCACTTACACAGGCCG | GAAGTCCACAGAGCCGTTCA |
| Ccl2 | Mouse | GGCTCAGCCAGATGCAGTTA | TTCCTTCTTGGGGTCAGCAC |
| Il1b | Mouse | TGCCACCTTTTGACAGTGATG | TGATGTGCTGCTGCGAGATT |

| | | | |
|---|---|---|---|
| Lcn2 | Mouse | CGAGTTACCTCGTCCGAGTG | CAGCCGTCGATACACTGGTC |
| Mmp9 | Mouse | GCAGAGGCATACTTGTACCG | TGATGTTATGATGGTCCCACTTG |
| Ptx3 | Mouse | CAGGAGAGCCGTGACGC | TGTTTCACAACCTGCGGGC |
| Stat2 | Mouse | TCCGCTGTTCGCTATCTTGG | TGCGCCATTTGGACTCTTCT |
| Tfpi2 | Mouse | AGCACTCTCCTCCCTCCAG | AGATCTCTAAATTATTCCCTTGGGC |
| Tnip1 | Mouse | CCTGGGCTGAAGCTAGGC | GTTCAAAAGCTGCGGACACC |
| Sod2 | Mouse | CAGACCTGCCTTACGACTATGG | CTCGGTGGCGTTGAGATTGTT |
| FNDC5 | Mouse | CTGTCTCCAATGTTCCACTTGTCTG | CTTGCCTTTGTTCTTTGAGGCCATC |
| Actb (β-actin) | Mouse | GGCTGTATTCCCCTCCATCG | CCAGTTGGTAACAATGCCATGT |
| Acta2 (a-SMA) | Mouse | GTCCCAGACATCAGGGAGTAA | TCGGATACTTCAGCGTCAGGA |
| Cnn1 (Calponin 1) | Mouse | TCTGCACATTTTAACCGAGGTC | GCCAGCTTGTTCTTTACTTCAGC |
| Myh11 (smMHC) | Mouse | AAGCTGCGGCTAGAGGTCA | CCCTCCCTTTGATGGCTGAG |
| Pecam1 (CD31) | Mouse | CTGCCAGTCCGAAAATGGAAC | CTTCATCCACCGGGGCTATC |
| Kdr (Flk-1) | Mouse | TTTGGCAAATACAACCCTTCAGA | GCAGAAGATACTGTCACCACC |
| Gata4 | Mouse | CCCTACCCAGCCTACATGG | ACATATCGAGATTGGGGTGTCT |
| Nkx2-5 | Mouse | TGACCCAGCCAAAGACCCT | CCATCCGTCTCGGCTTTGT |
| Nppa | Mouse | GCTTCCAGGCCATATTGGAG | GGGGGCATGACCTCATCTT |

## Supplementary References

[1] W. Yan, Y. Guo, L. Tao, W. B. Lau, L. Gan, Z. Yan, R. Guo, E. Gao, G. W. Wong, W. L. Koch, Y. Wang, X. L. Ma, *Circulation* **2017**, *136* (22), 2162.
[2] R. J. Vagnozzi, M. Maillet, M. A. Sargent, H. Khalil, A. K. Z. Johansen, J. A. Schwanekamp, A. J. York, V. Huang, M. Nahrendorf, S. Sadayappan, J. D. Molkentin, *Nature* **2020**, *577* (7790), 405.
[3] W. Yan, C. Lin, Y. Guo, Y. Chen, Y. Du, W. B. Lau, Y. Xia, F. Zhang, R. Su, E. Gao, Y. Wang, C. Li, R. Liu, X. L. Ma, L. Tao, *Circulation research* **2020**, *126* (7), 857.
[4] W. Yan, F. Zhang, R. Zhang, X. Zhang, Y. Wang, F. Zhou, Y. Xia, P. Liu, C. Gao, H. Wang, L. Zhang, J. Zhou, F. Gao, E. Gao, W. J. Koch, H. Wang, H. Cheng, Y. Qu, L. Tao, *Journal of molecular and cellular cardiology* **2014**, *74*, 183.
[5] E. J. Villablanca, J. R. Mora, *Journal of visualized experiments : JoVE* **2011**, (49).
[6] Y. Xia, F. Zhang, S. Zhao, Y. Li, X. Chen, E. Gao, X. Xu, Z. Xiong, X. Zhang, J. Zhang, H. Zhao, W. Wang, H. Wang, Y. Guo, Y. Liu, C. Li, S. Wang, L. Zhang, W. Yan, L. Tao, *Cardiovasc Res* **2018**, *114* (10), 1335.
[7] W. Yan, H. Zhang, P. Liu, H. Wang, J. Liu, C. Gao, Y. Liu, K. Lian, L. Yang, L. Sun, Y. Guo, L. Zhang, L. Dong, W. B. Lau, E. Gao, F. Gao, L. Xiong, H. Wang, Y. Qu, L. Tao, *Basic research in cardiology* **2013**, *108* (3), 329.
[8] X. Guan, W. Xu, H. Zhang, Q. Wang, J. Yu, R. Zhang, Y. Chen, Y. Xia, J. Wang, D. Wang, *Stem cell research & therapy* **2020**, *11* (1), 73.
[9] J. Pang, X. Qi, Y. Luo, X. Li, T. Shu, B. Li, M. Song, Y. Liu, D. Wei, J. Chen, J. Wang, C. Wang, *Theranostics* **2021**, *11* (5), 2381.
